# *In vivo* solid phase microextraction for therapeutic monitoring and pharmacometabolomic fingerprinting of lung during *in vivo* lung perfusion of FOLFOX

**DOI:** 10.1016/j.jpha.2023.04.005

**Published:** 2023-04-12

**Authors:** Nikita Looby, Anna Roszkowska, Miao Yu, German Rios-Gomez, Mauricio Pipkin, Barbara Bojko, Marcelo Cypel, Janusz Pawliszyn

**Affiliations:** a Department of Chemistry, University of Waterloo, 200 University Avenue West, Waterloo, ON, N2L 3G1, Canada; b Department of Pharmaceutical Chemistry, Medical University of Gdansk, 80-416, Gdansk, Poland; c Division of Thoracic Surgery, University Health Network, TGH, 200 Elizabeth St, Toronto, ON, M5G 2C4, Canada; d Department of Pharmacodynamics and Molecular Pharmacology, Collegium Medicum in Bydgoszcz, Nicolaus Copernicus University in Torun, 85-089, Bydgoszcz, Poland

**Keywords:** In vivo lung perfusion, Solid-phase microextraction, Chemotherapy, Metabolomics, Therapeutic drug monitoring

## Abstract

In vivo lung perfusion (IVLP) is a novel isolated lung technique developed to enable the local, in situ administration of high-dose chemotherapy to treat metastatic lung cancer. Combination therapy using folinic acid (FOL), 5-fluorouracil (F), and oxaliplatin (OX) (FOLFOX) is routinely employed to treat several types of solid tumours in various tissues. However, F is characterized by large interpatient variability with respect to plasma concentration, which necessitates close monitoring during treatments using of this compound. Since plasma drug concentrations often do not reflect tissue drug concentrations, it is essential to utilize sample-preparation methods specifically suited to monitoring drug levels in target organs. In this work, in vivo solid-phase microextraction (in vivo SPME) is proposed as an effective tool for quantitative therapeutic drug monitoring of FOLFOX in porcine lungs during pre-clinical IVLP and intravenous (IV) trials. The concomitant extraction of other endogenous and exogenous small molecules from the lung and their detection via liquid chromatography coupled to high resolution mass spectrometry (LC-HRMS) enabled an assessment of FOLFOX's impact on the metabolomic profile of the lung and revealed the metabolic pathways associated with the route of administration (IVLP vs. IV) and the therapy itself. This study also shows that the immediate instrumental analysis of metabolomic samples is ideal, as long-term storage at −80 °C results in changes in the metabolite content in the sample extracts.

## Highlights

• Use of in vivo SPME during FOLFOX administration in pre-clinical IVLP and IV trials.
• Therapeutic drug monitoring of FOLFOX and metabolites in porcine lung.
• Pharmacometabolomic fingerprinting of porcine lung during FOLFOX administration.
• *In vivo* SPME facilitates in situ monitoring of lung function with no tissue biopsy.

## Graphical abstract

**Figure:**
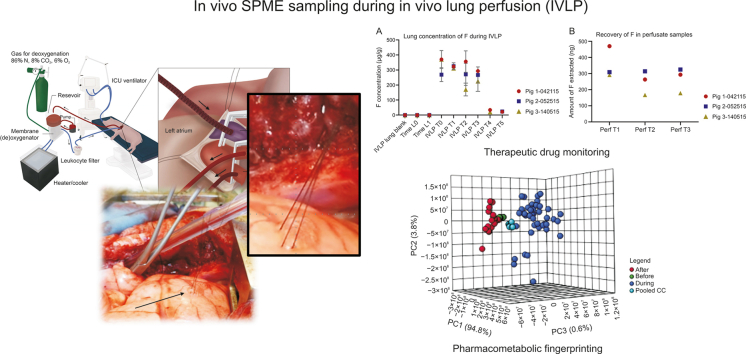

## 1 Introduction

Among the different combination chemotherapies used in oncology, the simultaneous administration of folinic acid (FOL), 5-fluorouracil (F), and oxaliplatin (OX) (FOLFOX) has proven to be effective for treating a wide range of solid tumours, including breast, head and neck, ovarian, colon, and metastatic lung cancers [[1], [2], [3], [4], [5]]. While FOLFOX-based chemotherapy is commonly administered intravenously (IV), this approach often produces side-effects due to drug-toxicity arising from systemic exposure to chemotherapy [4]. However, when treating terminal pulmonary diseases such as end-stage metastatic lung cancer, such risks can be minimized by using FOLFOX in conjunction with in vivo lung perfusion (IVLP). IVLP is a modified form of isolated lung perfusion—which was adapted from ex vivo lung perfusion—that can help facilitate the in situ treatment of terminal pulmonary diseases like end-stage metastatic cancer [5,6]. This targeted drug delivery system allows high doses of chemotherapy drugs such as FOLFOX to be administered exclusively to the lung, thereby minimizing systemic exposure to chemotherapy. Findings suggest that the use of IVLP in conjunction with metastasectomy, currently the most effective treatment for lung cancer metastases, can help to reduce disease recurrence, as it can eliminate any undetected, and therefore unresected, micrometastases remaining in the peripheral tissue following tumor resection [5].

Each of FOLFOX's constituent drugs possesses complex mechanism of action that produces multiple metabolites. For instance, FOL-related metabolites include 5,10-methylenetetrahydrofolate (5,10-methyleneTHF), 5-methyltetrahydrofolate (5-methylTHF), tetrahydrofolate (THF), while F-related metabolites include 5-fluorodeoxyuridine (5FdUrd), 5-fluorodeoxyuridine monophosphate (5FdUMP), 5-fluorodeoxyuridine triphosphate (5FdUTP), 5-fluorouridine (5FUrd), 5-fluorouridine triphosphate (5FUTP) [2,4,7], dihydrofluorouracil (FUH_2_), fluoroureidopropionic acid (FUPA), and fluoro-beta-alanine (FBAL) [1,4,7]. OX, which is a third-generation platinum-based chemotherapy drug [8] possessing various mechanisms of action, has been suggested to reduce F catabolism by inhibiting dihydropyrimidine dehydrogenase enzyme activity, thereby contributing to the synergistic effects of FOLFOX-based chemotherapy [1].

Although several studies have established that FOLFOX provides satisfactory antitumor activity, the development of standard dosing methods has been limited, as high inter-patient variability in blood plasma concentrations of this drug can result in over- or under-exposure [4,9,10]. Personalized approaches based on the monitoring of concentrations of these drugs during treatment can maximize therapeutic outcomes and minimize adverse effects by reducing the chances of over- or under-dosing. Therapeutic drug monitoring (TDM) is employed to measure concentrations of drugs in biological matrices, such as blood and its fractions (i.e., plasma or serum) or tissue, following administration. TDM is required when using drug combinations like FOLFOX, as these drugs are characterized by narrow therapeutic ranges and large inter-patient variability [10]. Although it is relatively easy to monitor concentrations of these drugs in plasma, this approach is not always suitable because plasma concentrations may not reflect those in the specific target tissues or organs. This further exacerbates challenges related to TDM for tissue analysis [11]. In clinical settings, TDM on tissue matrices requires multiple invasive biopsies to be performed over the course of treatment. Additionally, the sample-preparation procedures required to analyze these biopsies, such as cryogenic pulverization or other homogenization methods combined with solid-liquid extraction (SLE), are typically time-consuming, laborious, and require multiple steps [11].

Metabolomics is an emergent “omics” strategy in systems biology that has been proposed as an alternative approach to assessing the efficacy of (combination) chemotherapy treatments [12]. Briefly, metabolomics entails the study of all metabolites in a given system at a given time [13,14]. The use of metabolomics or pharmacometabolomics—the study of the metabolome in response to pharmaceuticals—to monitor one or a multitude of metabolic biomarkers for a particular chemotherapy dose or treatment can yield vital information about drug resistance, the development of drug resistance, and overall positive, negative, or indifferent responses to treatment [12]. While metabolomics approaches generally remain difficult to implement due to challenges associated with identifying and validating biomarkers, validating the methods used to obtain these biomarkers, and correctly interpreting candidate biomarkers, the application of such approaches could nevertheless guide treatment and provide information about patient responses [12].

Despite the availability of targeted approaches like TDM and the potential of untargeted approaches like metabolomics, it remains difficult to obtain precise information about drug concentrations and the efficacy of combination therapy, especially during IVLP in lung tissue. Another challenge in employing metabolomics is the need to ensure the system under study is not disturbed by external stimuli, such as tissue biopsy. However, biopsies involve removing a piece of the organ from the bulk, which can lead to changes in the metabolic profile of the collected lung sample, as well as changes in the metabolic profile of the bulk itself due to injury sustained during the biopsy. In the case of TDM during IVLP, current tissue-based drug-determination methods only utilize lung biopsies collected prior to the start of IVLP, and at the end of IVLP after the lung has been reconnected for systemic circulation and reperfusion has been sustained for at least 2 h. In the post-IVLP case, the lung is sufficiently flushed of any drugs before being reconnected to systemic circulation, thus resulting in drug concentration levels that do not reflect those present during IVLP. Furthermore, biopsies are taken from peripheral locations rather than the bulk of the lung, which typically experiences higher rates of perfusion. Thus, existing tissue-sampling protocols are insufficient for accurately determining drug concentrations in the lung during IVLP. Nonetheless, it is critical to define the relationship between the administered chemotherapeutic dose and the effective concentration in the sampled biofluid and/or tissue.

One method potentially capable of overcoming the challenges associated with TDM, metabolomics, and tissue analysis is in vivo solid-phase microextraction (SPME), which is a novel, non-exhaustive sample-preparation approach that performs extraction from the free concentration of analytes. In vivo SPME, a small needle (200 μm in diameter) coated with a biocompatible polymeric extraction phase (40 μm thickness) is introduced into the solid tissue (e.g., lung), thus eliminating the need for biopsy collection [[15], [16], [17], [18]]. SPME's simple design and negligible depletion of the system under study not only facilitate in vivo sampling, thereby overcoming an inherent limitation of other tissue-sampling techniques, but it also amalgamates sampling and sample-preparation into a single step. The polyacrylonitrile-based extraction phase allows the diffusive partitioning of a broad range of freely-available small-molecule compounds (<1000 Da) from complex biological matrices onto the SPME coating, thereby enabling the extraction and monitoring of all small molecules, such as drugs and endogenous metabolites [19,20]. When coupled with liquid chromatography (LC) and highly specific and sensitive detectors, such as tandem mass spectrometry (MS/MS) or high resolution mass spectrometry (HRMS), SPME enables the targeted and untargeted (metabolomics) analysis of all compounds isolated from the biological tissue [18]. Furthermore, SPME can be easily coupled directly to mass spectrometry via a microfluidic open interface to enable rapid therapeutic and point-of-care analysis of drugs or metabolites in typically challenging matrices (e.g., tissues), especially under in vivo conditions [21,22].

In the present study, SPME is proposed as an in vivo sampling technique for the simultaneous therapeutic monitoring of FOLFOX and its metabolites in pig lungs during the pre-clinical IVLP and IV administration of this drug. The chemotherapeutic efficacy of these administration routes was subsequently assessed via pharmacometabolic fingerprinting using liquid chromatography  coupled to high resolution mass spectrometry (LC-HRMS) to illustrate SPME's ability to characterize changes in lung tissue over the course of the IVLP and IV procedures. Furthermore, a supplementary study was conducted to assess the metabolomic profile's stability under the standard storage conditions currently employed in metabolomics. The objective of this supplementary study was to determine whether these standard storage conditions (−80 °C) were conducive to reliable sample preservation.

## 2 Materials and methods

### 2.1 Chemicals and standards

**Fig. S1:**
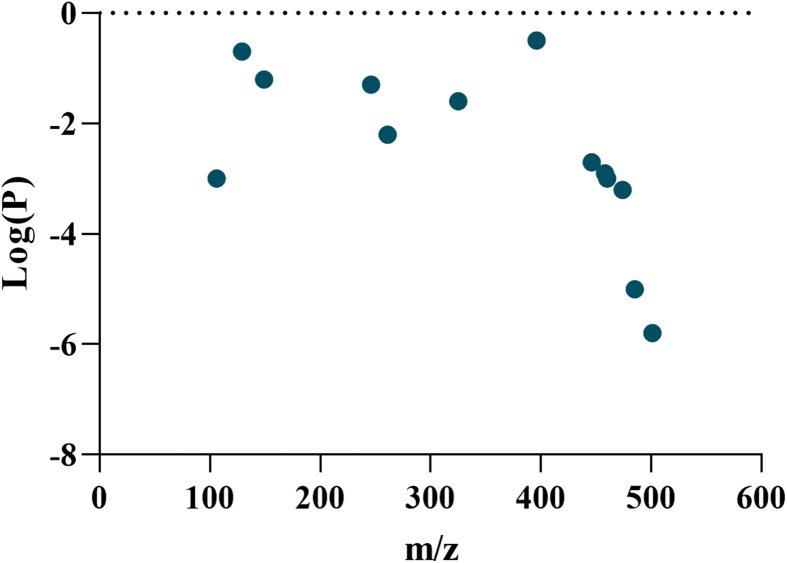
Distribution of FOLFOX compounds and their metabolites according to their mass-to-charge ratio (*m*/*z*) and physiochemical properties (LogP). The provided log (P) values are a mix of experimental and predicted values obtained from DrugBank [32].

(6R,S)-5,6,7,8-tetrahydrofolic acid hydrochloride (THF) and 5FdUrd were purchased from Cayman Chemical Company (Ann Arbor, MI, USA), while Uridine-^15^N_2_5′-monophosphate (UMP-IS), uridine^15^N_2_5′-triphosphate (UTP-IS), FOL (leucovorin) calcium, and F were purchased from Sigma-Aldrich (St. Louis, MO, USA). The 5FdUTP, 5FdUMP, and 5FUTP used in this work were purchased from Sierra Bioresearch (Tuscon, AZ, USA), and the 5,10-methylTHF, 5-methylTHF, folic acid-d2 (FA-d2), FUPA, ureidopropionic acid (FUPA-IS), FBAL, alpha-fluoro-beta-alanine-^13^C_3_ (FBAL-IS), 5-fluorouridine-^13^C,^15^N_2_ (5FUrd-IS), 5-fluoro-2′-deoxyuridine-^13^C,^15^N_2_ (5FdUrd-IS), OX, carboplatin, and chlorouracil were purchased from Toronto Research Chemicals (Toronto, Canada). The log (P) values of FOLFOX compounds and their metabolites are provided in Fig. S1. Finally, LC-MS-grade solvents, including acetonitrile, water, and methanol, and LC-MS-grade additives, including formic acid, acetic acid, and ammonium acetate, were all purchased from Fisher Scientific (Ottawa, Canada).

### 2.2 Animals and research ethical approval

Four Yorkshire pigs with an average weight of 35 kg were used for the IVLP (3 cases) and IV (1 case) experiments. This study was approved by the Institutional Review Board (IRB) at the University Health Network (UHN; Toronto, ON, Canada) and the University of Waterloo’s Research Ethics Board (# 40573), and all animals received humane care in compliance with the Principles of Laboratory Animal Care outlined by the National Society for Medical Research and the Guide for the Care of Laboratory Animals instituted by the National Institutes of Health.

### 2.3 IVLP procedure

The IVLP procedure used in this work has been described in detail elsewhere [5,6]. Briefly, after the induction of general anaesthesia, the left pulmonary artery (PA) and the left pulmonary veins (PV) were dissected and isolated via a left thoracotomy, with ventilation to the left lung being maintained by an intensive care unit ventilator. After the administration of heparin, the left PA and PVs were cannulated and clamped before initiating IVLP in order to isolate the left lung from systemic circulation. 1.2 L of Steen™ solution/perfusion fluid (XVIVO Perfusion, Gӧteburg, Sweden) was then circulated through the isolated organ using a centrifugal pump. The perfusion fluid was first passed through a membrane gas exchanger connected to a heater/cooler, which was responsible for maintaining it at normothermic conditions; the warmed solution was then passed through a leucocyte filter, entered the lung via the left PA, and exited through the left PV into a hard-shell reservoir that was kept at a certain height to ensure suitable PA and PV pressures. Upon entering the reservoir, the fluid was then recirculated through the circuit. Drug doses were calculated based on the pigs' body weights and body surfaces areas using Brody's formula [23]. 400 mg/m^2^ of FOL was administered intravenously. Once maximum circuit flow was reached, FOX was administered directly into the perfusion circuit through the reservoir as a bolus at a concentration of 400 mg/m^2^ for F, and concentrations of either 255 mg/m^2^ or 170 mg/m^2^ for OX. After 4 h of IVLP, a 10 to 15 min flushing step was conducted using 500 mL of pure perfusate (Perfadex; XVIVO Perfusion), followed by the removal of the cannulas from the PA and PVs. The lung was then reconnected to systemic circulation and reperfusion (normal systemic circulation) was initiated for a 2 h period.

### 2.4 IV procedure

A left thoracotomy was performed to allow the sampling fibers access to the lung; however, no further surgical processes took place. FOLFOX was administered intravenously at 400 mg/m^2^ for FOLF and 85 mg/m^2^ for OX for a period of approximately 2 h.

### 2.5 Lung sampling protocol

**Fig. 1:**
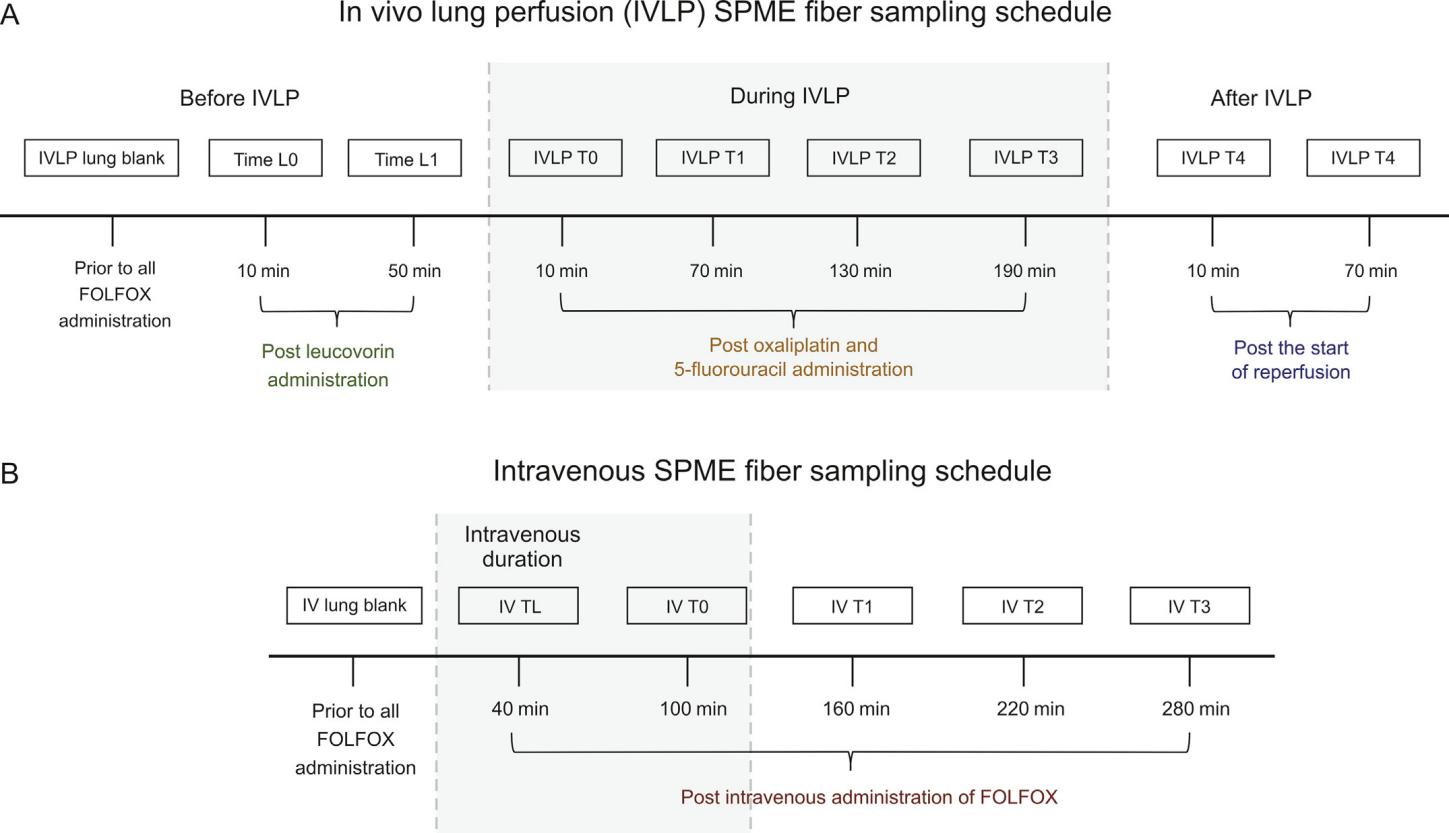
Lung sampling schedule using solid-phase microextraction (SPME) fibers during (A) in vivo lung perfusion (IVLP) (top schedule) and (B) intravenous (IV) (bottom schedule) administration of folinic acid (FOL), 5-fluorouracil (F), and oxaliplatin (OX) (FOLFOX).

The SPME fibers used to perform in vivo lung sampling in the IVLP and IV cases contained an octadecyl-strong-cation-exchange (C_18_-SCX) coating and were kindly provided by Supleco (Mississauga, Canada). The fibers were initially sterilized in a solvent mixture consisting of methanol:water (50:50, *V/V*) for a minimum of 30 min. Following sterilization, the fibers were removed from the conditioning solvent and inserted into the lung in triplicate approximately 1–2 cm apart at predetermined time points (shown in Fig. 1 and described in more detail below). After the extractions had been completed, the fibers were removed from the lung tissue, rinsed manually in water for 5 s to remove any loosely adhered biological matter, wiped with a Kimwipe, and placed in empty 300 μL polypropylene vials. The vials were then snap frozen in dry ice for transportation to the lab. The fibers remained stored in the empty vials at −80 °C until the time of instrumental analysis. The experiment was conducted in accordance with the procedure outlined by Roszkowska et al. [24]. For more information on the in vivo SPME method developed for the quantification of FOLFOX in porcine lung tissue, please refer to in vivo SPME method development (Supplementary data).

The left lung was sampled according to the schedule outlined in Fig. 1A. Briefly, the lung was initially sampled at baseline (IVLP lung blank) post sternotomy, and prior to the addition of FOLFOX; it was then sampled 10 min after the IV administration of FOL (Time L0), and again 60 min after the initial administration of FOL (Time L1). Due to surgical time restrictions, a 10 min extraction time was used for each of these three sampling points prior to IVLP. The next round of sampling was conducted immediately after the introduction of FOX into the perfusion circuit and 10 min (IVLP T0) after the commencement of IVLP (full flow established), with subsequent samplings taking place at 1-h intervals during the 4 h of perfusion (IVLP T1-IVLP T3), and again at 1-h intervals during the 2 h of reperfusion (IVLP T4-IVLP T5). An extraction time of 30 min was employed for sampling during IVLP and reperfusion.

During the IV administration of FOLFOX, the lung was sampled according to the schedule outlined in Fig. 1B. Briefly, the lung was sampled at baseline (IV lung blank) prior to the administration of FOLFOX, and again at 40 min (IV TL) after the start of the bolus administration of FOLFOX. The lung was subsequently sampled hourly at 100 min, 160 min, 220 min, and 280 min (IV T0–IV T3).

### 2.6 Perfusate sampling protocol

Single perfusate samples were collected in parallel with the IVLP sampling schedule outlined in Fig. 1A following the same protocol that was used for lung sampling. C_18_-SCX fibers were sterilized/conditioned in methanol:water (50:50, *V/V*) and then exposed to 300 μL of perfusion fluid for a total of 30 min under static conditions. The fibers were then removed, rinsed manually for 5 s in pure water, and stored in empty 300 μL vials, which were snap frozen in dry ice and stored at −80 °C until further analysis. The procedure employed to prepare the fibers for lung and perfusate sampling is described in detail in in vivo SPME method development (Supplementary data).

### 2.7 Sample preparation and sample extracts

Sampling and sample preparation, which are condensed into a single step in SPME, were conducted at the hospital during the pre-clinical IVLP and IV trials. Just prior to instrumental analysis, the fibers were removed from storage at −80 °C and desorbed at room temperature in 300 μL of an acetonitrile:water (50:50, *V/V*) solution for 60 min at 1500 rpm. At the end of the 60 min desorption period, LC-HRMS was used to perform metabolomic analysis on the final solvent extracts. FOLFOX levels were quantitated using a method developed retroactively after the samples had been collected, wherein a portion of the extracts were transferred from their original vials (20 μL) to separate vials and mixed with 6 μL of an IS mixture to produce a final concentration of each IS at 200 ng/mL. Quantitative FOLFOX analysis and screening for FOLFOX metabolites was then performed by subjecting these newly prepared solutions to LC-MS/MS. More information on the LC-MS/MS method developed for quantitative analysis of FOLFOX from porcine lung tissue can be found in LC-MS/MS method development (Supplementary data).

### 2.8 Stability and storage conditions

All samplings were performed at Toronto General Hospital in 2015. Upon the completion of each sampling, the fibers were snap frozen in dry ice and stored in empty 300 μL vials for transportation to the laboratory where they were stored on fiber at −80 °C until instrumental analysis. Following the desorption processes, and after LC-HRMS instrumental analysis, the sample extracts were re-stored at −80 °C until they were analyzed again two years later in 2017. These sample extracts were consistently stored under the above conditions and were not subjected to multiple freeze-thaw cycles (less than two cycles).

### 2.9 LC-HRMS for untargeted analysis

Pharmacometabolic fingerprinting was performed on the porcine lung and perfusate extracts collected during the pre-clinical IVLP and IV trials using a Thermo Exactive orbitrap mass analyzer mass spectrometer (Waltham, MA, USA) coupled to an Accela binary pump and autosampler. Chromatography was completed on a Discovery® HS F_5_-3 column (100 mm × 2.1 mm, 3 μm particle size; MilliporeSigma, Bellefonte, PA, USA) protected by a Discovery® HS F_5_-3 guard column (20 mm × 2.1 mm, 3 μm particle size), using a 40 min method that had previously been developed in laboratory [25,26]. Mobile phases A and B consisted of water and acetonitrile, respectively, with 0.1% formic acid added to each for positive mode analysis, and 0.1% acetic acid added to both for negative mode analysis. The column was maintained at 30 °C, and the samples were kept in the autosampler at 4 °C. A pooled quality control (QC) consisting of 10 μL of each sample extract was collected in a separate vial and injected every 10 samples during instrumental acquisition. An injection volume of 10 μL was used for LC-HRMS analysis.

### 2.10 LC-MS/MS method for FOLFOX and metabolites screening

The chromatographic separation of F, 5-FUrd, 5-FdUrd, 5-FUTP, 5-FdUTP, 5-FdUMP, FBAL, FUPA, OX and their respective ISs was performed using a Sequant® Zic®-pHILIC column (100 mm × 2.1 mm, 5 μm particle size; MilliporeSigma, Darmstadt, Germany) protected by a corresponding Sequant® Zic®-pHILIC column guard column (20 mm × 2.1 mm, 5 μm particle size). LC-MS/MS was conducted using a Thermo Vanquish quaternary pump and an autosampler coupled to a Thermo TSQ Vantage triple quadrupole mass spectrometer equipped with an Ion Max™ atmospheric pressure ionization (API) heated electrospray source (HESI) (Waltham, MA, USA). Water (mobile phase A) and acetonitrile (mobile phase B), each with 0.1% acetic acid and 5 mM ammonium acetate, were employed at a total flow rate of 300 μL/min. An injection volume of 5 μL was utilized and the samples and column were maintained at 5 °C and 25 °C, respectively. The above-noted compounds were ionized in negative mode at 2 kV in position D. The full chromatographic method is outlined in Tables S1–S4. HESI source parameters, MS parameters, information on the LC gradient and information on the development of the LC-MS/MS method used in this work are described in detail in LC-MS/MS method development (Supplementary data).

**Fig. S2:**
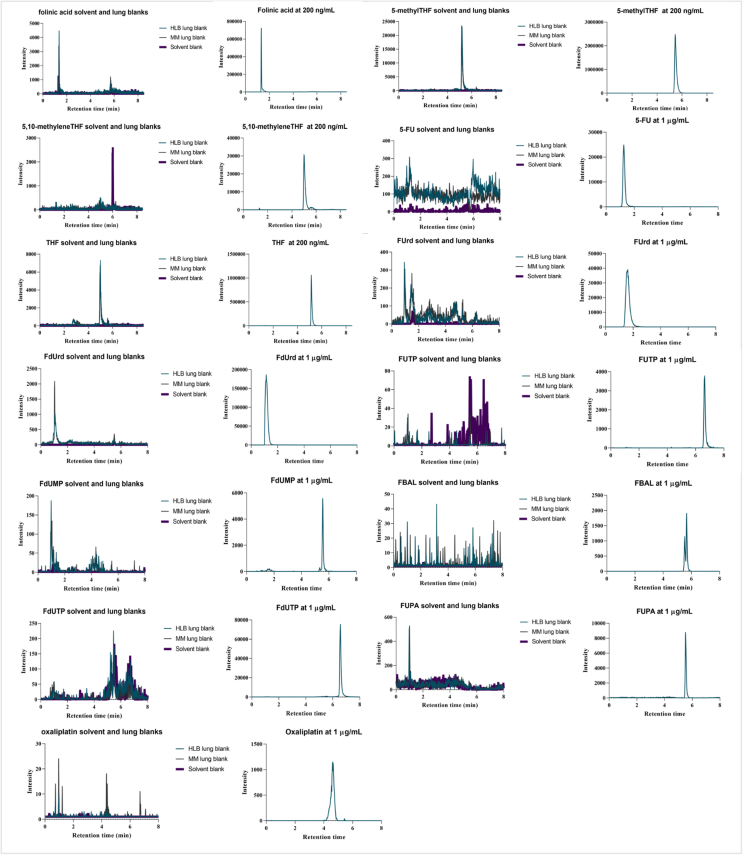
Evaluation of LC-MS/MS selectivity and specificity. Neat solvent blank and extracts obtained from blank (non-spiked) lung homogenate with the HLB and C_8_-SCX (mixed-mode, MM) coatings were injected and monitored at the mass spectrometric transitions of the target analytes. The blank signal from the HLB coating is shown in turquoise, the signal from MM is shown in dark gray, and the signal from the neat solvent is shown in dark purple bolded.

The separation of FOL, 5,10-methylTHF, 5-methylTHF, THF, and folic acid-d2 was achieved using a Discovery® HS F_5_-3 (PFP) column (100 mm × 2.1 mm, 3 μm particle size; MilliporeSigma, Bellefonte, PA, USA) protected by a corresponding Discovery® HS F_5_-3 guard column (20 mm × 2.1 mm, 3 μm particle size) as part of the above-described LC-MS/MS system. Here, mobile phases A and B consisted of water and acetonitrile, respectively, with 0.25% acetic acid and 0.05% formic acid added to each. Furthermore, these separations utilized the same flow rate and autosampler conditions described above, a column temperature maintained at 30 °C, and an injection volume of 10 μL. The compounds were ionized in positive mode at 1.3 kV in position B. The chromatograms illustrating the LC-MS/MS method's specificity and selectivity can be seen in Fig. S2 (data shown for HLB- and C_8_-SCX-coated fibers). More information relating to the MS parameters, LC gradient, and HESI source parameters is provided in LC-MS/MS method development (Supplementary data).

### 2.11 Final calibration curve, quality control, and quantitation

**Fig. S3:**
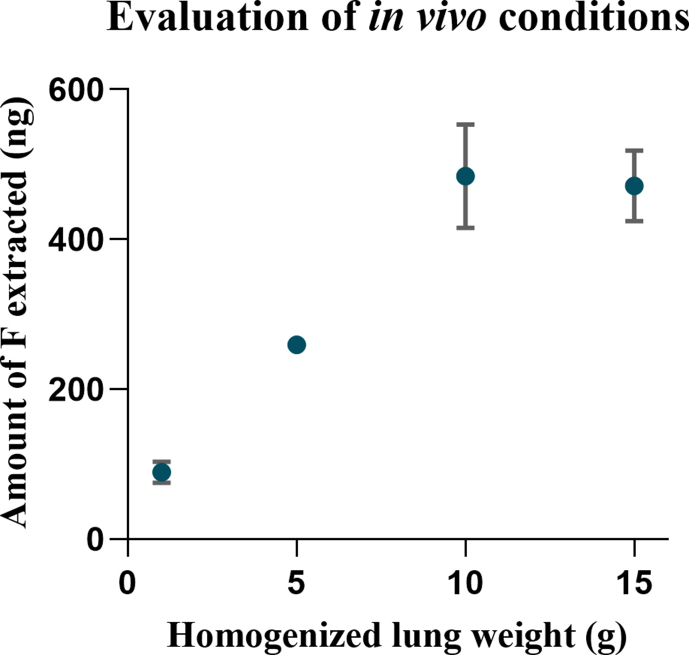
The assessment of in vivo conditions for appropriate calibration. The following weights of homogenized lamb lung were tested: 1, 5, 10, and 15g. This test sought to find the conditions under which the amount of extracted F becomes independent of the sample weight.

**Fig. S4:**
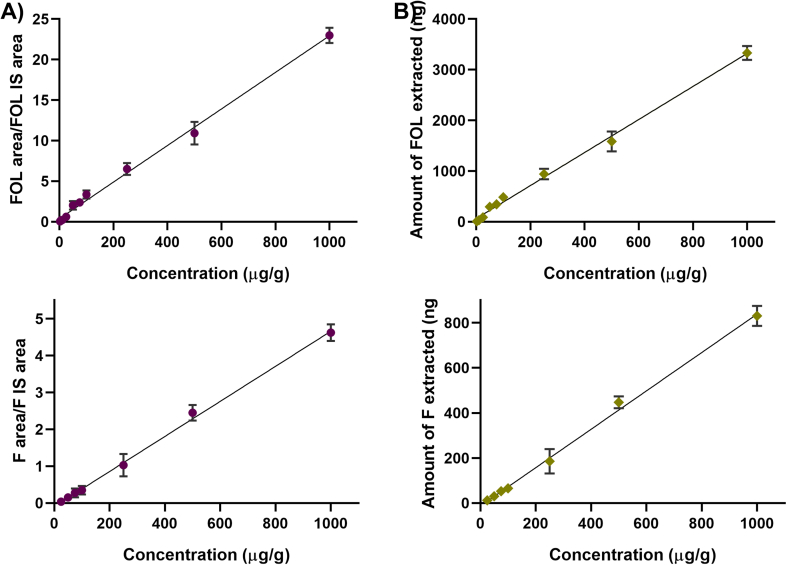
Matrix-matched external calibration curve in lamb lung homogenate for FOL (A) and F (C). A linear dynamic range of 50 μg/g to 1000 μg/g with a linearity (R^2^) of 0.99 was achieved for both compounds. Each calibration curve was correlated with the amount of extracted FOL (B) and F (D) to streamline the analytical process. Linear dynamic ranges corresponding to 295 ng–3000 ng and 30 ng–800 ng were achieved for FOL and F, respectively.

The in vivo study conditions possessed inherent limitations that precluded the use of pre-loaded ISs on the SPME fiber. As such, kinetic calibration methods using an IS could not be applied for quantitation. Instead, a matrix-matched external calibration curve was constructed using 15 g of lamb lung homogenate (Fig. S3) containing a mixture of analytes at concentrations ranging from 2 μg/g to 2,000 μg/g. ISs were only added to a portion (20 μL) of the lung sample extracts after desorption step, thus correcting for LC-MS injection during metabolomic analysis, but not for the extraction process. The matrix-matched external calibration curve was then cross-referenced against to an instrumental curve that ranged from 0.001 to 1 μg/mL for F and 0.01 to 3.5 μg/mL for FOL to minimize the use of large amounts of lung homogenate and to simplify the quantitation process. Ultimately, a limit of detection (LOD) of 25 μg/g and a limit of quantitation (LOQ) of 50 μg/g were achieved for both F and FOL (Fig. S4). Quality control samples containing FOL and F at concentrations of 100 μg/g and 500 μg/g were internally assessed by back calculating the validated matrix-matched lung homogenate calibration curve with these points excluded. More information regarding the preparation of the calibration curve and method validation can be found in in vivo SPME method development (Supplementary data).

### 2.12 Data pre-processing for untargeted analysis

After instrumental analysis via LC-HRMS, the raw files were converted to mzXML files using MSConvert. Initially, the IPO package from the XCMS software package was applied using a script developed in-laboratory in RStudio to optimize the peak-picking parameters based on the pooled QCs [[27], [28], [29]]. These optimized parameters were then applied to perform data pre-processing functions, such as noise filtering and baseline correction, peak detection and deconvolution, retention time and mass-to-charge ratio (*m/z*) alignment and correction. The final data matrix, which contained all sample names and all features with their respective retention times and intensities across the samples, was filtered by eliminating features in the pooled QCs with relative standard deviations (RSD) > 30% and a pooled QC-to-blank ratio < 5. The xMSAnnotator Integrative Scoring Algorithm was employed to annotate features from each group in the data with respect to retention time clusters, adduct formation, isotope patterns, and abundance and pathway analysis [30]. Only high- and medium-confidence matches with unique or multiple feature identities were considered for further data interpretation. Tables S5–S7 present annotated features of interest that changed over the course of IVLP in the lung (Table S5) and in perfusate (Table S6), as well as in the lung during IV administration (Table S7).

### 2.13 Chemometric analysis and model validation

Statistical analysis, including multivariate and univariate analysis, was performed using Metaboanalyst 3.0. The quality of the instrumental run was initially assessed by conducting principal component analysis (PCA) on the structure of the pooled QCs, which was followed by the application of partial least squares-discriminant analysis (PLS-DA) and orthogonal projection to latent structures discriminant analysis (O-PLS-DA) to fingerprint/classify the clustered groups. Univariate analysis via the Kruskal-Wallis test was employed to identify any features that changed significantly (false discovery rate adjusted *P* < 0.05) across two or more time points during IVLP or IV. Only models that passed across validation were further considered. The PLS-DA model generated for the sampling time points during IVLP (IVLP T0 to IVLP T3) was validated using a permutation test (at 1000) and 10-fold cross validation (CV). The model passed both CVs, with the original data classification generating a significant difference (*P* < 0.01) in comparison to the remaining permuted data distribution, while the 10-fold CV produced a model fit of *R*^2^ = 0.90 and a predictability of Q^2^ = 0.79. Therefore, variables of importance to projection (VIP) with scores greater than 1.5 (VIP > 1.5) were further investigated and tentatively identified using METLIN and cross referenced based on annotation. Features of interest tentatively and putatively identified with a mass accuracy of less than 5 ppm are listed in Table S8.

## 3 Results and discussion

### 3.1 Analysis of porcine lung extracts obtained from pre-clinical trials of FOLFOX chemotherapy using IVLP and IV

**Fig. S5:**
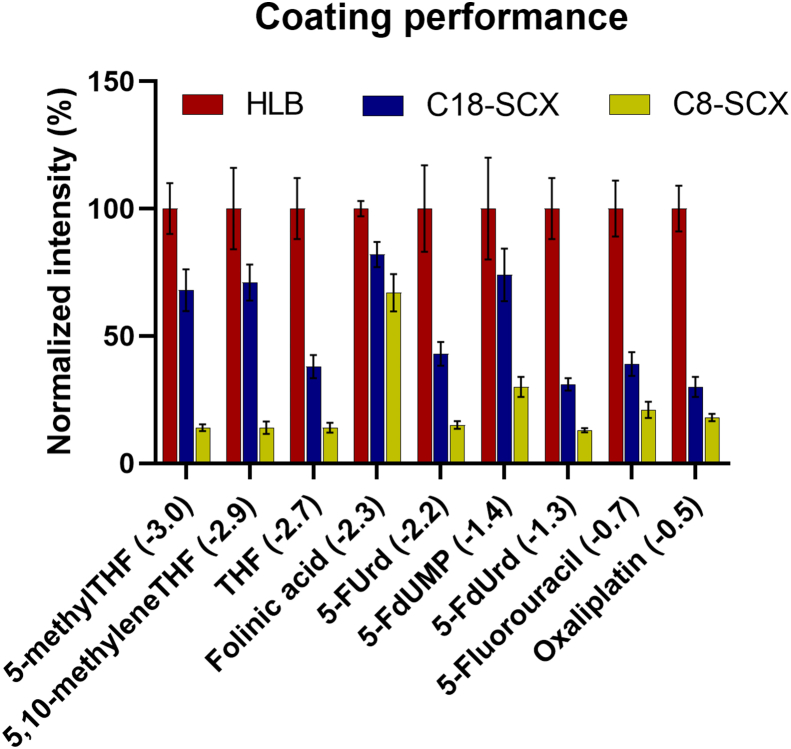
Evaluation of coating performance based on extractions from PBS spiked at clinically expected concentrations. Values were normalized based on the intensity observed with HLB. The HLB coating is shown in red, the C_18_-SCX is shown in blue, and the C_8_-SCX coating is shown in yellow. The relative recoveries are shown for each compound along with their log P values in parentheses.

**Fig. S6:**
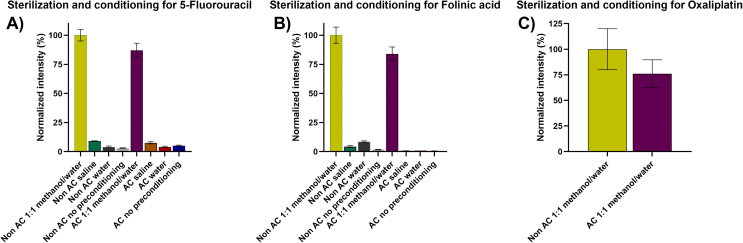
Evaluation of sterilization and preconditioning of C_8_-SCX-coated fibers for (A) F, (B) FOL, and (C) OX. Sterilization by autoclave was tested against non-autoclaved fibers. For each category of steam sterilization, preconditioning solutions of 50:50 methanol/water, saline solution, pure water, and no solution were tested.

**Fig. S7:**
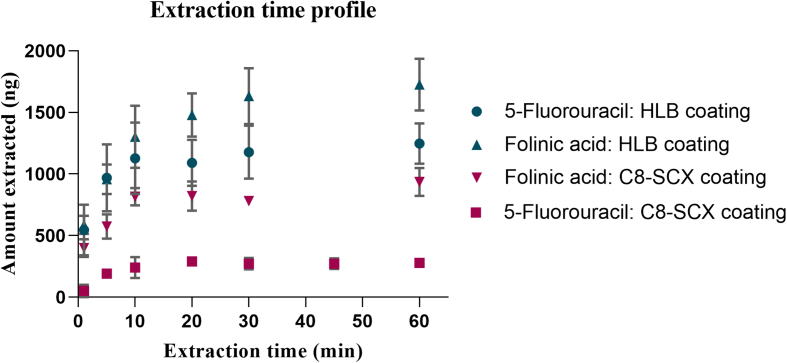
Extraction time profile of FOLFOX from lamb lung homogenate. The following extraction times were tested: 1, 5, 10, 20, 30, 45, and 60 min. The HLB and C_8_-SCX coatings were evaluated using 5 replicates for each coating at each time point.

**Fig. S8:**
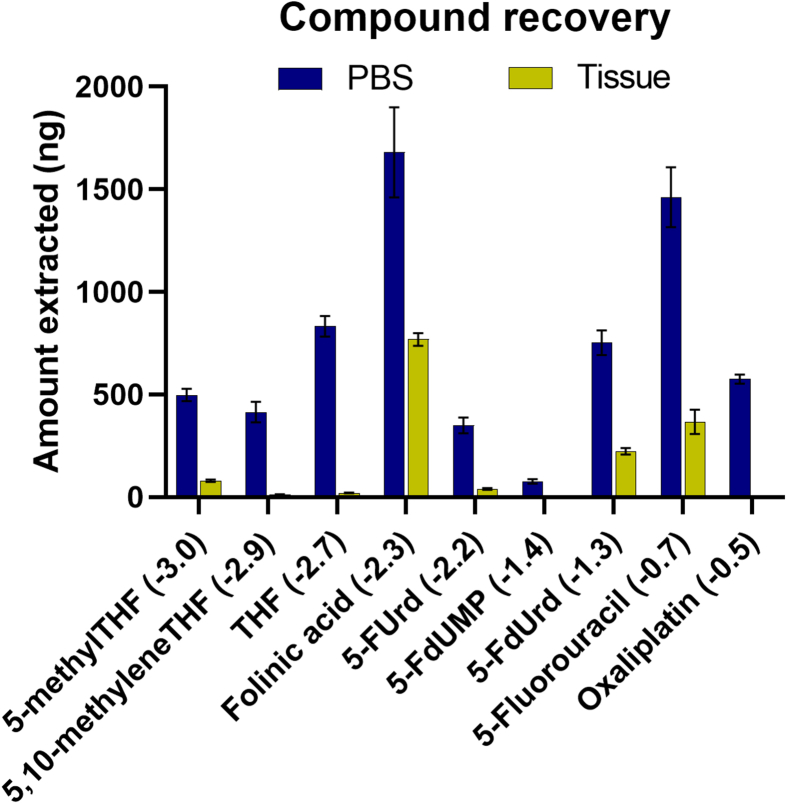
Absolute recoveries of FOLFOX and its metabolites from phosphate buffered saline (PBS) and homogenized lamb lung tissue spiked at clinically expected concentrations. The log P value for each compound is provided in parentheses. Results shown only for the C_8_-SCX coating.

The application of the retrospective SPME-LC-MS/MS method revealed that the hydrophilic-lipophilic balance (HLB) coating provided the best sensitivity for the compounds and metabolites of interest (Fig. S5). However, the tests conducted during method development also showed that C_18_-SCX-coated fibers, which were used during the pre-clinical IVLP and IV trials, were rather suitable as well. In addition, preconditioning in methanol:water (50:50, *V/V*) was determined to be an acceptable sterilization method for non-autoclaved SPME devices, as it enabled the maintenance of good coating extraction efficiency (Fig. S6). Furthermore, an extraction time of 30 min, which was used during sampling prior to method development, was also found to be within equilibrium conditions for the investigated HLB and octyl-strong cation exchanger (C_8_-SCX) coatings, with the supposition that the C_18_-SCX coating would achieve equilibrium within comparable times (Fig. S7). Nonetheless, since the C_8_-SCX-coated fibers were used to build the calibration curve and the C_18_-SCX-coated fibers were used to perform sampling, it is only possible to form a close estimate of FOLFOX concentrations in the collected pre-clinical samples, while allowing for a 20%–50% difference in these obtained values for FOL and F. These limits are based on the differences observed in the recovery of these compounds during the coating performance tests described in in vivo SPME method development (Supplementary data). The negligible recovery of OX was initially contributed to high binding and rapid non-enzymatic degradation (Fig. S8), since OX is known to have a half-life of only 14 min in biological systems [31]. As a result, OX as a whole drug subsequently could not be fully recovered in neither tissue nor under aqueous conditions like phosphate buffered saline (PBS). Considering the 3-h equilibration time after spiking the lung tissue with FOLFOX, the results for OX correlate with the experimental conditions. It is important to emphasize, however, that SPME can still extract OX under the appropriate conditions, such as those during the IVLP and IV trials.

**Fig. 2:**
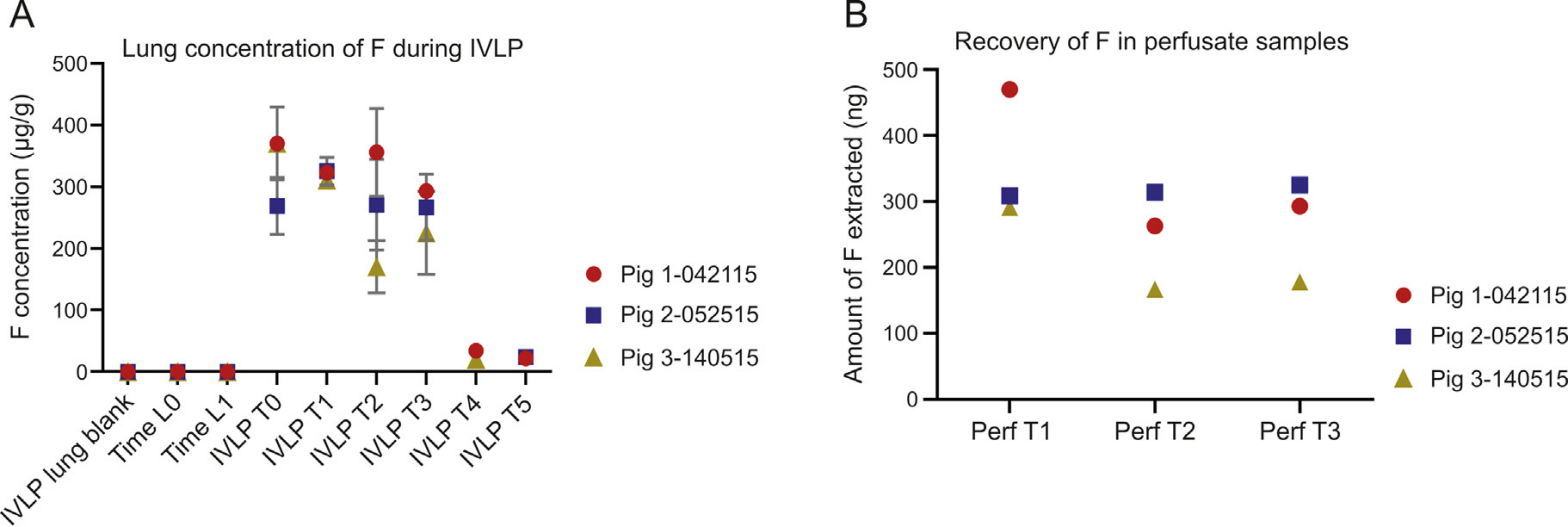
Analysis of 5-fluorouracil (F) in pre-clinical in vivo lung perfusion (IVLP) lung sample extracts using the in vivo solid-phase microextraction coupled to liquid chromatography with tandem mass spectrometry (SPME-LC-MS/MS) method. (A) Lung concentration of F throughout the IVLP procedure. (B) Absolute recovery of F obtained from perfusate (Perf) samples collected during IVLP.

In all three pre-clinical IVLP cases, no FOLFOX or metabolites were detected in any of the pig lung blanks, and F was quantifiably recovered only in samples collected after the administration of FOX and during IVLP from T0 to T3 (Fig. 2A). As Fig. 2A shows, F appears to maintain a steady concentration range of 250 μg/g to 350 μg/g throughout IVLP. This trend could be indicative of F's low protein binding of 8%–12% (information provided by DrugBank) [32], which leaves much of the drug available in free form during IVLP. The larger variances observed for sampling points are likely related to the non-homogenous distribution of this drug in the lung. This steady-state trend is mirrored in the results for the perfusate samples shown in Fig. 2B. Notably, the amount of available F appears to drop at Perf T2 and Perf T3 for Pigs 1 and 3 (compared to Perf T1), while no such drop is observable for Pig 2. This might be the result of different rates of enzymatic activation or degradation among the pigs; however, it is difficult to reliably conclude what this trend signifies, as only one perfusate sample was taken at each time point. Given the differences in the devices used for calibration (C_8_-SCX) and sample collection (C_18_-SCX), the actual concentration is likely twice the above-reported values, or between 500 μg/g and 700 μg/g. Trace amounts of F were detected in the samples collected during the 2 h of reperfusion, which suggests that performing a flushing step with fresh perfusion fluid prior to reperfusion prevents systemic exposure to high drug concentrations. While 5FdUrd and 5FUrd were also detected in the lung samples, they were largely found in perfusate samples on an inconsistent basis. For example, 5FdUrd was detected in one pig's lung as early as 1 h after the start of IVLP (IVLP T0) and approximately 30 min after the administration of FOX, but was not detected in the other two pigs at the same time point. Furthermore, in all pigs, 5FdUrd could be detected at IVLP T1, but was undetectable by IVLP T3. Conversely, 5FUrd was mainly detected at IVLP T2, IVLP T3, and, in some cases, IVLP T4 and IVLP T5 for pigs 1 and 2. Traces of 5FdUTP and 5FUTP were also observable in a few perfusate and lung samples from as early as IVLP T1 to as late as IVLP T5. The presence of these metabolites indicates that the metabolism of F into active metabolites is an ongoing process. On the contrary, neither F nor its metabolites were found in the lungs during the IV case using the developed LC-MS/MS method. FBAL and FUPA were not detected in any of the samples in the IVLP and IV cases, which may be the result of various factors such as the poor analyte affinity for the coating, as demonstrated in the coating performance tests, rather than an indication of their absence in lung tissue. Additionally, this result may also be due to the weak circulation of F metabolites into the lung, or the lack of F metabolite formation in the liver in the case of IV drug administration.

**Fig. 3:**
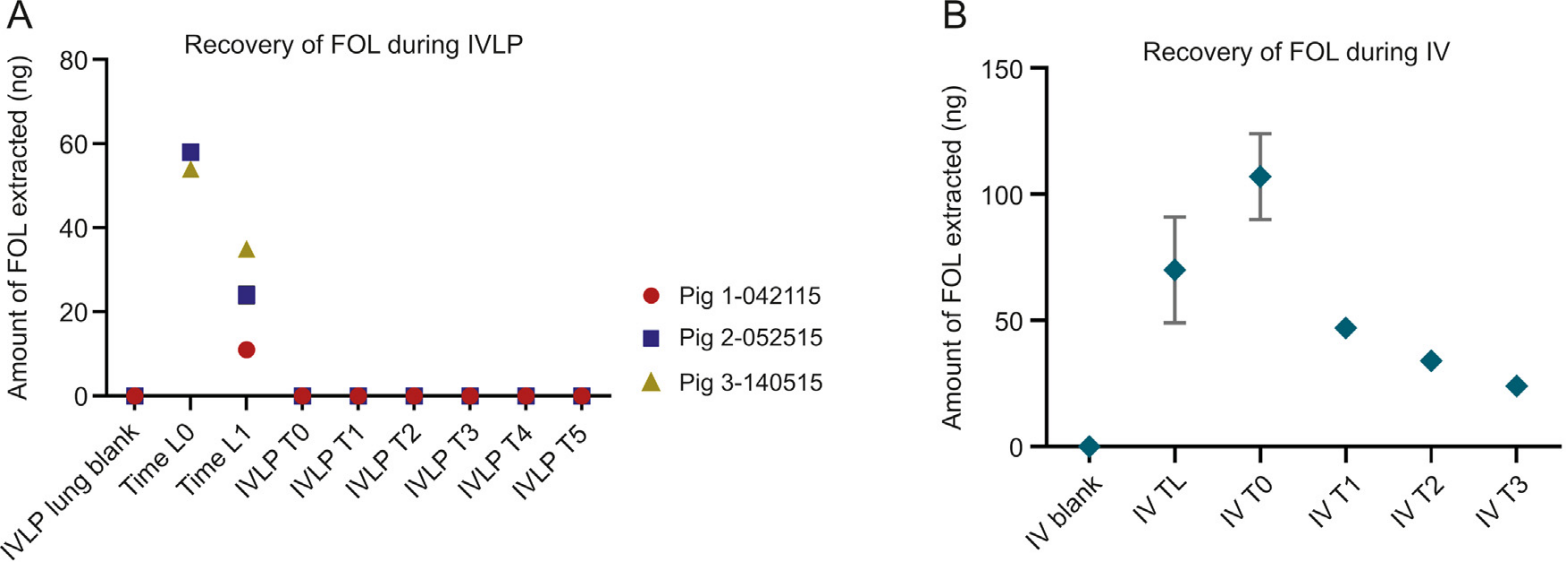
Analysis of folinic acid (FOL) in pre-clinical in vivo lung perfusion (IVLP) lung sample extracts using the in vivo solid-phase microextraction coupled to liquid chromatography with tandem mass spectrometry (SPME-LC-MS/MS) method. (A) Absolute recoveries of FOL from lung tissue during IVLP. (B) Absolute recoveries of FOL from lung tissue during intravenous (IV) administration of folinic acid, 5-fluorouracil, and oxaliplatin (FOLFOX).

FOL was detectable (Fig. 3A) at 10 min (Time L0) and 50 min (Time L1) after its initial IV administration. However, the amount of FOL recovered from the lung was inconsequential, falling below the 25 μg/g LOQ for its determination in tissue (interpolated to be between 5 and 25 μg/g). Thus, the concentration of FOL could not be reliably quantified with this method, and the amount recovered (in ng) was used instead (Fig. 3B). A decrease in the amount of FOL recovered in the IVLP cases was observed over the 60 min interval from Time L0 to Time L1 (Fig. 3A). A similar trend was also observed during the IV administration case (Fig. 3B), with the FOL concentration appearing to peak 100 min after the commencement of FOLFOX infusion (IV T0) and then slowly decreasing. This trend suggests that the FOL was either partitioning into cells or undergoing metabolism. It should be noted that 5-methylTHF has been reported as the final product of FOL metabolism, and has been shown to be the most stable of all FOL-related metabolites, especially under a wide pH range (pH 2–10) [[33], [34], [35]]. While 5,10-methyleneTHF was not detected, 5-methylTHF was detected at Time L0 and Time L1 in the IVLP cases. Similarly, 5-methylTHF was also detected throughout the IV cases from as early as IV T0, all the way through to 100 min after FOL administration and at peak FOL concentration, which supports the observation of rapid drug metabolism. However, it is also important to consider the tendency of 5,10-methyleneTHF to rapidly convert to 5-methylTHF under various temperatures and pH conditions. As such, one cannot rule out the possibility that this transformation could occur during sample storage, as no antioxidants were being added to the sample extracts. Therefore, given the weak stability of FOL metabolites, it would be difficult to accurately quantify the absolute amounts of each of these compounds in biological material, and only the report of their existence during metabolite screening was possible.

### 3.2 Untargeted pharmacometabolomic analysis

To monitor the biochemical profile of the lung during IVLP and IV FOLFOX-based chemotherapy, SPME was also applied to isolate other endogenous and exogenous small molecules from the lung tissue and perfusate samples at the pre-determined sampling points. The molecular features (metabolites) detected in the SPME extracts were identified via annotation. SPME was coupled with LC-HRMS, with the results revealing changes in the metabolomes of the porcine lungs and perfusate (Tables S5–S7).

**Fig. 4:**
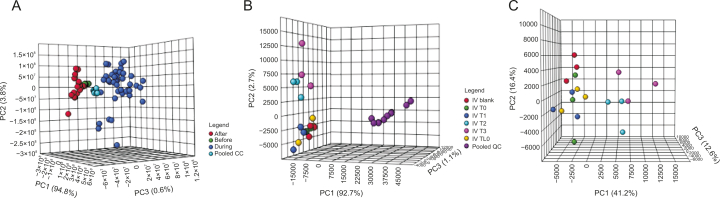
The principal component analysis (PCA) plots of the overall structure of the data for lung samples extracts taken during in vivo lung perfusion (IVLP) and intravenous (IV) administration of folinic acid, 5-fluorouracil, and oxaliplatin (FOLFOX). (A) PCA (PC1: 94.8%, PC2: 3.8%, PC3: 0.6%) plot for lung sampling events over the course of IVLP. Samples taken before, during, and after IVLP (during reperfusion) are represented in green, blue, and red, respectively. Pooled quality controls (QCs) are represented in turquoise. (B) PCA (PC1: 92.7%, PC2: 2.7%, PC3: 1.1%) plot of lung sampling events during IV administration. Samples collected at baseline and hourly for 5 h after the start of infusion are shown in red, yellow, green, blue, turquoise, and pink, respectively. Pooled QCs are represented in purple. (C) PCA (PC1: 41.2%, PC2: 16.4%, PC3: 12.6%) plot for samples shown in (B) with pooled QCs removed to enable closer observation.

In examining the overall data structure of the samples, which were initially analyzed in 2015 and again in 2017, it is evident that the instrumental analysis in both cases was reliable, as the pooled QCs clustered well in the centre of all the samples on the PCA plot (Figs. 4A and B for IVLP and IV, respectively). Analysis of the three biological replicates (3 pigs sampled) included in the analysis and the three technical replicates (3 fibers) collected at each sampling time for each pig clearly revealed a significant difference between the samples, specifically for the lung sampling events. This difference can be seen in the categorical clustering of each group in Fig. 4A: the samples collected prior to the start of IVLP (lung blanks and samples collected after FOL administration) are shown in green, those collected during IVLP (IVLP T0 to IVLP T3) are shown in blue, and those collected during reperfusion after IVLP (IVLP T4 and IVLP T5) are shown in red. Interestingly, contrary to expectations, there was no clustering corresponding to the progression of the IVLP procedure from the first hour to the fourth hour. However, the IV case from the same period (2015) (Figs. 4B and C with pooled QCs removed) shows distinct clustering of the lung samples collected prior to FOLFOX administration (IV Blank–represented in red) and clear separation between these samples and the last two sampling time points (IV T2 and IV T3), with overlap occurring between the three interim sampling time points (IV TL0, IV T0, and IV T1).

Compositional changes in the endogenous metabolites in the lung tissue observed over the course of IVLP were related to the metabolism of amino acids, acylcarnitines, purines and pyrimidines, lipids, and pro- and anti-inflammatory compounds, among others (Table S5). In addition, these metabolomic alterations were also reflected in the composition of the perfusate samples (Table S6). For instance, the number of amino acids initially detected in the lung tissue significantly decreased approximately 60 min after the administration of FOL (Time L1 in Fig. 1A) and remained low until the end of the reperfusion period, whereas the analysis of the perfusate samples collected during IVLP revealed a higher number of free amino acids (Table S6). Conversely, the systemic administration (IV) of FOLFOX did not significantly affect the number of amino acids, as the composition of these compounds remained relatively constant in the lung tissue for more than 4 h after the IV administration of FOLFOX (Table S7). Furthermore, after IV administration, the total number of detected free amino acids was much higher compared to the number of these compounds extracted from the lungs during IVLP, which may be due to the differences in the action of drugs administered directly to the lungs and via systemic circulation.

Although low numbers of several lipid species were detected early in the IVLP procedure, including bioactive lipids (oxylipids) with either pro- or anti-inflammatory activity, the number of these compounds significantly increased by the second hour post FOX administration (IVLP T2) (Table S5). These alterations were even more profound in the perfusate samples (Table S6). Interestingly, a tremendous release of pro- and anti-inflammatory compounds was also observed in the lung tissue in the IV case approximately 3 h (IV T1) post FOX administration (Table S7). This result strongly indicates that, for oxylipins specifically, the changes with respect to pro- and anti-inflammatory compounds in lung tissue over time are more related to the applied polytherapy than the route of drug administration (IVLP vs. IV).

Apart from the above-mentioned group of compounds, the overall metabolomic profile of lung tissue after the IV administration of FOLFOX differed significantly from the lung tissue profile observed during IVLP. For instance, a group of endogenous compounds known as autacoids—specifically, resolvins and neuroprostanes—were detected in lung tissue and perfusate samples solely during the IVLP procedure (i.e., 60 min post FOX administration). These locally acting compounds possess potent immunoregulatory properties and may strongly affect the activity of the immune system [36]. Interestingly, these autacoids were not detected in lung tissue after the IV administration of FOLFOX, which indicates that FOX's distinct impact on the metabolomic profile of lung tissue—and, consequently, the pharmacokinetics and pharmacodynamics of the polytherapy in the targeted organ—is strongly dependent on the administration route. It should also be noted that the concentration of metabolites, or intermediates of purine and pyrimidine metabolism, remained relatively constant in lung tissue after the systemic administration of FOLFOX (Table S7), but decreased significantly during the local administration of FOLFOX via IVLP (Table S5).

### 3.3 Storage conditions and metabolite stability

**Fig. 5:**
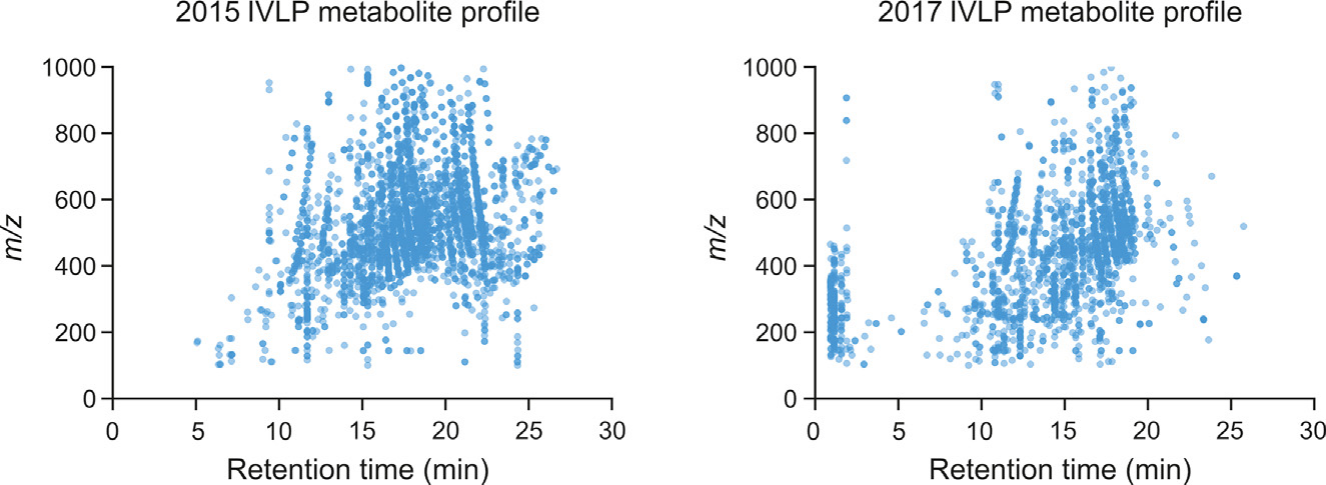
(A) Metabolite profile of pre-clinical in vivo lung perfusion (IVLP) lung sample extracts obtained and analyzed via liquid chromatography coupled to high resolution mass spectrometry (LC-HRMS) in 2015. (B) Metabolite profile of the same pre-clinical IVLP lung sample extracts upon re-analysis in 2017. Both sets of data were filtered in the same way.

**Fig. 6:**
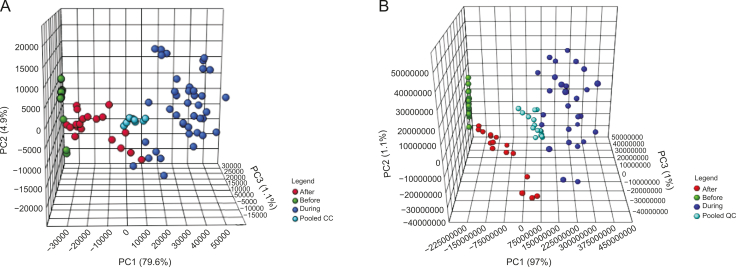
The liquid chromatography coupled to high resolution mass spectrometry (LC-HRMS) of lung samples taken before the start of in vivo lung perfusion (IVLP) (IVLP Lung Blank, Time L0, Time L1), during IVLP (IVLP T1 to IVLP T3), and post IVLP during reperfusion (IVLP T4 and IVLP T5). Each group of samples is represented by green, red, and blue, respectively. (A) PCA (PC1: 79.6%, PC2: 4.9%, PC3: 1.1%) of samples acquired in 2017. (B) PCA (PC1: 97%, PC2: 1.1%, PC3: 1%) of the same samples in 2015. Each data set shows the clustering of the pooled quality controls (QCs) at the center of the data (represented by turquoise).

Fig. 5 shows that approximately 40% of the information contained in the samples was lost during storage, which corresponds to nearly 1400 features. While the PCAs obtained from both sets of data exhibit a similar structure (Fig. 6), the metabolite profiles for the lung samples before, during, and after IVLP are noticeably different (Fig. 5). For example, a significant loss of features within a large *m/z* range between retention times of 20 min and 27 min can be observed from 2015 (Fig. 5A) to 2017 (Fig. 5B). Additionally, the PCAs also show a loss in the number of features greater than 600 *m/z* between retention times of 10 min and 13 min and the appearance of features below 500 *m/z* with retention times of less than 3 min, which could be products of degradation. While it is difficult to pinpoint what causes these changes during storage under these conditions, the profiles clearly show that data integrity degrades over prolonged storage times. Given the time-consuming nature of data processing and statistical analysis in metabolomics, it may be quite challenging to employ tandem mass spectrometry for metabolite validation if discriminating features or features of importance have degraded. Indeed, as was observed in this study, analysis performed before and after storage showed drastic differences in the same data sets for samples taken before, during, and after IVLP. Moreover, annotation of the 2017 data revealed almost no information of importance due to the loss of almost half the data initially annotated in 2015. While the annotated peaklist from the 2017 data showed that most amino acids were retained in the data from 2015, it also indicated the presence of only a few peptides, thus suggesting the degradation of peptides during sample storage. In addition, various lipids species were detected in small numbers in the 2017 data (vs. the 2015 data), indicating the substantial degradation of these compounds during storage.

## 4 Conclusions

In this study, SPME was successfully applied for the repeated and easy in vivo sampling of complex biological matrices, including porcine lung, during the pre-clinical IV and IVLP administration of FOLFOX chemotherapy. Following SPME sampling, comprehensive quantitative and qualitative separation methods were employed to determine FOLFOX concentrations in lung tissue, screen for FOLFOX metabolites, and perform general pharmacometabolic fingerprinting. This work is the first documented attempt to develop a complementary, all-encompassing LC-MS/MS method for the quantitation and screening of FOLFOX and its metabolites. Additionally, while both FOLFOX administration methods appeared to produce metabolites that were indicative of the therapy's effectiveness, the level of these compounds in the lungs varied depending on the selected route of administration (IVLP vs. IV). Furthermore, the results of the supplementary study, which evaluated the metabolome stability of extracts stored at −80 °C over a long period of time (2 years), illustrate the challenges associated with metabolomic studies, as the observed loss of data highlights the importance of performing analysis and MS/MS validation as soon as possible after sample collection. Overall, this study successfully demonstrated SPME's effectiveness as an in vivo sampling tool for use in the concomitant quantitative monitoring of polychemotherapy and qualitative lung assessment via metabolite screening and pharmacometabolomic fingerprinting during the IVLP and IV administration of FOLFOX.

## CRediT author statement

**Nikita Looby**: Methodology, Validation, Formal analysis, Investigation, Data curation, Writing - Original draft preparation, Reviewing and Editing, Visualization; **Anna Roszkowska**: Methodology, Investigation, Data curation, Writing - Original draft preparation, Reviewing and Editing; **Miao Yu**: Software, Validation, Writing - Reviewing and Editing; **German Rios-Gomez**: Conceptualization, Methodology, Data curation; **Mauricio Pipkin**: Methodology, Validation, Investigation, Writing - Reviewing and Editing; **Barbara Bojko**: Conceptualization, Writing - Reviewing and Editing, Project administration, Funding acquisition; **Marcelo Cypel**: Conceptualization, Resources, Supervision, Writing - Reviewing and Editing, Project administration, Funding acquisition; **Janusz Pawliszyn**: Conceptualization, Resources, Supervision, Writing - Reviewing and Editing, Project administration, Funding acquisition.

## Declaration of competing interest

The authors declare that there are no conflicts of interest. The information included in this manuscript are a part of PhD thesis of Dr Nikita Looby, which was published by the University of Waterloo (https://uwspace.uwaterloo.ca/bitstream/handle/10012/15892/Looby_Nikita.pdf?sequence=5&isAllowed=y).
