# Supplementary material for: In vivo solid phase microextraction for therapeutic monitoring and pharmacometabolomic fingerprinting of lung during in vivo lung perfusion of FOLFOX
Source: J Pharm Anal. 2023 Apr 12;13(10):1195–204. doi: 10.1016/j.jpha.2023.04.005 (PMC10657970; doi:10.1016/j.jpha.2023.04.005)
Supplement: Multimedia component 1 [file mmc1.docx]

**Supplementary Information**

**In vivo solid-phase microextraction for therapeutic monitoring and pharmacometabolomic fingerprinting of lung during in vivo lung perfusion of FOLFOX**

**LC-MS/MS method development**

1. LC method development

Before a targeted liquid chromatographic tandem mass spectrometric (LC-MS/MS) method can be developed, it is important to consider all the compounds of interest and their subsequent numerous metabolites that result of undergoing various biochemical processes. Folinic acid (FOL) and be converted to 5,10-methylenetetrahydrofolate (5,10-methyleneTHF), 5-methyltetrahydrofolate (5-methylTHF), tetrahydrofolate (THF) while oxaliplatin (OX) undergoes non-enzymatic degradation. Fluorouracil (F) however, can be converted to useful drug metabolites such as 5FdUMP), 5-fluorodeoxyuridine triphosphate (5FdUTP), 5-fluorouridine (5FUrd), 5-fluorouridine triphosphate (5FUTP) or broken down to ineffective metabolites like dihydrofluorouracil (FUH2), fluoroureidopropionic acid (FUPA), and fluoro-beta-alanine (FBAL). While, 5-fluorouracil (F), and oxaliplatin (OX) (FOLFOX) compounds are used in combination with one another for chemotherapy, the physicochemical properties of these compounds and their numerous metabolites possess a wide range of mass as well as polarities. These characteristics ultimately influence their isolation from complex biological matrices and their separation and ionization on any analytical platform, especially liquid chromatography coupled to mass spectrometry (LC-MS). The log (P) values provided in Fig. S1 are a mix of experimental and predicted values obtained from DrugBank. Generally, these compounds have been ionized exclusively in either positive (OX, FOL, and FOL metabolites) or negative mode (F and F metabolites), which effectively renders the use of a single LC-MS/MS method impractical unless instruments capable of polarity switching are utilized. Furthermore, various column types have been successfully used for separation of only subsets of these compounds, thus requiring the use of three or more LC-MS/MS methods for the screening and/or quantitation of all FOLFOX drugs and drug metabolites. To address this challenge, several columns were evaluated during the development of an LC method that could encompass all the target compounds (FOLFOX drug and drug metabolites). The following column were thus investigated: a Hypercarb column (100 mm x 2.1 mm, 5µm particle size) (Thermo Scientific, Waltam, MA, USA), a Kinetex pentafluorophenyl (PFP) column (100 mm x 2.1 mm, 2.6 µm particle size) (Phenomenex, Torrance, CA, USA), a Discovery^®^ HS F_5_-3 (PFP) column (100 x 2.1 mm, 3 µm particle size) (Millipore Sigma, Bellefonte, PA, USA), an Atlantis C_18_ column (70 mm x 2.1 mm, 5 µm particle size) (Waters Corporation, Milford, MA, USA), and a Sequant^®^ Zic®-pHILIC column (100 mm x 2.1 mm, 5 µm particle size) (MilliporeSigma, Darmstadt, Germany). Acetonitrile (ACN), water, and methanol were tested as mobile phases, while formic acid, acetic acid, ammonium acetate, ammonium formate, purchased from Fisher Scientific (Ottawa, Canada) or a mixture thereof were tested as mobile phase additives.

The final chromatographic conditions for FOX and metabolites utilized a zic-pHILIC column are as outlined in Table S1. The MS parameters and conditions used for the analysis of FOX and F metabolites can be found in Table S2. The final chromatographic conditions for FOL and FOL metabolites utilized a Discovery (PFP) column. The chromatographic conditions and MS parameters employed for their analysis is listed in Table S3 and Table S4, respectively.

2. Mass spectrometric parameters and conditions

FOL, 5,10-methyleneTHF, 5-methylTHF, and THF were tuned and analyzed in positive mode, while oxaliplatin, F, 5FdUMP, 5FdUTP, 5FUTP, 5FUrd, 5FdUrd, FUPA, FUH2 and FBAL were tuned and analyzed in negative mode. All compounds were initially tuned at a concentration of 1 µg/mL in ACN:water (50:50, v/v) solution containing 0.1% acetic acid, with flow rates of 5 µL/min and 7-10 µL/min for positive and negative mode ionization, respectively. For poor ionizers, infusion was instead performed at either 5 µg/mL or 10 µg/mL. The tuning parameters and conditions are outlined in Table S2 and Table S4.

3. Selectivity and specificity of LC-MS/MS method

To ensure that the developed LC-MS/MS method was free of interferences from co-extracted or co-eluted endogenous compounds, solid phase microextraction fibers that were used for in vivo lung sampling and for SPME-based method development (octadecyl-strong cation exchanger (C_18_-SCX), hydrophilic-lipophilic balance (HLB) and octyl-strong cation exchanger (C_8_-SCX)) were used to perform extractions from blank (non-spiked) homogenized lamb lung tissue that had been used to prepare the matrix-matched external calibration curve. In addition, neat solvent blanks were also tested. Furthermore, fibers inserted into the lung at baseline during the experiments were also used as a blank control to evaluate interferences from the live matrix in vivo. Neither the solvent blanks nor the blank homogenate lung extractions showed any significant interferences for the specific transitions of any of the desired target compounds or metabolites. The chromatograms illustrating the LC-MS/MS method’s specificity and selectivity can be seen in Fig. S5 (data shown for HLB- and C_8_-SCX-coated fibers).

**In vivo SPME method development**

1. Fiber coating procedure

HLB and C_8_-SCX fibers were investigated during the retrospective SPME method development as alternative and competitive extraction phases to C_18_-SCX fibers. The latter was used for lung sampling during preclinical IVLP and IV trials but were later discontinued and replaced by newly manufactured C_8_-SCX fibers. Thus, these two coatings were worth exploring since C_8_-SCX was the functional replacement and HLB has been reported to be particularly useful for isolating compounds characterized by low logP values. To this end, an in-house developed coating procedure was used to coat nitinol wires with 5 µm HLB particles (kindly provided by Waters Corporation (Milford, MA, USA) until the coating dimensions measured 1.5 cm in length and 40 µm in thickness. Briefly, this dip-coating method utilized an automated, software-operated program to dip the 4 cm nitinol wires into a homogenously mixed slurry composed of 10 % (w/v) particles, 1 % glycerol (v/v), and 89 % (v/v) of 7 % (w/v) polyacrylonitrile (PAN). This dipping process was repeated until the desired coating thickness had been achieved.

2. Coating performance and absolute recoveries

At the inception of the study this method was developed for, the clinical application of SPME was rather novel. Moreover, access to various coating types and in-house coating protocols were limited at the time, which led to the exclusive use of LC- and bio-compatible C_18_-SCX-coated fibers that were also newly commercially available. Given that this study involved both targeted and untargeted analyses, these devices were deemed ideal, as they featured good inter-fiber reproducibility, which is essential for reliable data collection, especially for metabolomics. Unfortunately, as mentioned above, production of these C_18_-SCX coatings was halted near the end of the study and were replaced by octyl-strong-cation-exchange (C_8_-SCX) coatings. This posed a problem, as there were very few remaining C_18_-SCX fibers available for extensive method development. As such, a retrospective evaluation was completed to compare these newly available C8-SCX coatings against both the previously employed C_18_-SCX coating and a newly developed (at the time) HLB-coated devices that could be prepared in-laboratory.

The performances of all three coatings were evaluated via recovery tests in PBS, with the results shown in Fig. S6. The absolute recoveries from PBS and homogenized tissue were only tested for HLB and C_8_-SCX due to the limited number of C_18_-SCX fibers remaining (results in Fig. S6 shown only for C_8_-SCX as these were the commercially available replacements for C_18_-SCX). PBS and tissue were spiked at clinically expected concentrations for FOL, F, and OX (FOLFOX), while all associated metabolites were spiked at an arbitrary concentration less than the clinically expected concentrations to verify that they can be captured by SPME. PBS was spiked at 300 µg/mL and 100 µg/mL for FOLFOX and the metabolites, respectively, while lamb lung homogenate was spiked at 300 µg/g for FOLFOX and 100 µg/g for the metabolites. Five replicates were used to perform extraction for each coating at each time point.

The HLB coating exhibited superior recovery for most of the compounds (Fig. S7), a result that is consistent with previous findings showing its excellent extraction capabilities, especially for very polar compounds like FOL, F and OX from aqueous matrices. HLB showed as little as 1.5 times improvement in extraction efficiency compared to C_18_-SCX, and as much as 10 times improvement for some compounds compared to C_8_-SCX. Interestingly, C_18_-SCX demonstrated the next best recovery for these compounds, followed by its replacement C_8_-SCX. This trend is likely due to the strong interactions between the polar moieties of the target compounds and metabolites and the polar N-vinylpyrrolidone group on the HLB coating. Furthermore, it also appears that many of these compounds and metabolites contain aromatic or ring groups that better interact with the divinylbenzene group on the HLB coating via dispersion forces. This speculation is supported by the lower recoveries for slightly smaller but comparatively linear metabolites, such as FUPA and FBAL (results not shown), which would have less overall interaction due to their decreased molecular surface area. Thus, weak interaction between short hydrocarbon chains and aromatic extraction phases could explain the decreased recovery of these compounds via HLB. Moreover, the greater recovery for the majority of compounds and metabolites with the C_18_-SCX coating, as opposed to its short-chain counterpart, C_8_-SCX, supports the deduction that extraction efficiency is influenced by the degree of surface area interaction between the compounds and the coatings. While this would suggest improved recoveries for ring-containing metabolites with larger molecular weights, such as 5-FdUTP and 5-FUTP, the observed negligible recoveries (results not shown) could also be due to slower diffusion kinetics since the PBS extractions were performed under static conditions, or simply that these compounds are extremely polar with logP values of less than -5.0 and have a very low affinity (partition coefficient) for the coating. The coating performance trends observed in tissue homogenate mirrored those observed in PBS, with HLB providing better extraction efficiency and coverage of compounds than C_8_-SCX. However, the much lower recovery rates from homogenized tissue compared to PBS (Fig. S6) suggest significant matrix binding for many analytes, which therefore affects the free concentration available for SPME extraction. It is important to emphasize that SPME performs extraction via free concentration. This means that only a fraction of the unbound portion of the total amount of drugs or metabolites present in a system, is available for extraction by the device. This explains the significantly lower recovery from tissue vs. PBS. Additionally, for some metabolites that were already difficult to extract from PBS, such as 5FdUTP, 5FUTP, FUPA, and FBAL due to their polarity or structure, failed to be extracted altogether even when spiked at the same concentration as the parent compound F, a concentration which is much higher than the expected metabolite concentration.

3. FOLFOX compounds and drug metabolites stability

The OX, F, and F metabolites investigated in this study have previously been reported as being stable in solvent and in extracts from a range of biological matrices, including plasma, cell lines, and tissue. As such, the stability of these metabolites was not evaluated further in this study. Since SPME provides quenching by exclusively extracting small molecules from very complex matrices via free concentration while selectively excluding red blood cells, macromolecules such as proteins, and other large molecules (> 1000 Da). The direct desorption of the fibers into the desorption solvent is comparable to the storage of each compound or metabolite under solvent conditions. For example, dihydropyrimidine dehydrogenase (DPD), the major enzyme that catabolizes F, is found in red blood cells and tissues. However, SPME does not extract red blood cells or tissue fragments; rather, its PAN-based coating essentially acts as a high-molecular-weight sieve that allows it to extract only small molecules available in free concentrations. Thus, the extracted drugs and metabolites desorbed into the desorption solution will behave similarly to how they would in pure solvent, as they will not undergo enzymatic degradation. However, folates such as FOL and FOL metabolites are prone to rapid degradation in light, air, and under various pH conditions and temperatures; therefore, these compounds require the presence of an antioxidant in the solution. Thus, 1% ascorbic acid was added to the 50:50 acetonitrile/water desorption solution used throughout the development of the proposed method.

4. Extraction time profile

30 min was the pre-determined extraction time for sampling during the pre-clinical IVLP and IV trials. It is important, however, to determine whether this 30-min extraction time falls under the pre-equilibrium, kinetic or equilibrium conditions of the time profile in order to appropriately account for any reproducibility issues that may result from slight variations during the active sampling protocol as a result of unforeseen surgical circumstances. As such, an extraction time profile was constructed using lamb lung homogenate in order to investigate the time at which the equilibrium was achieved for the HLB and C_8_-SCX coatings (Fig. S8). Extraction times of 1, 5, 10, 20, 30, 45 and 60 min were selected given the time limitations imposed by the surgical procedure. After being exposed to the lung homogenate for the respective extraction times, the fibers were removed, manually rinsed in 300 µL of water for 5 s, wiped with a Kimwipe, and then desorbed in 100 µL of 50:50 acetonitrile/water containing 200 ng/mL of internal standards.

The extraction conditions used in this retrospective analysis were set to closely mimic clinical conditions in terms of the drug concentration administered into the perfusate. Therefore, lamb lung homogenate was spiked at concentrations of approximately 300 µg/g and 170 µg/g for FOLF and OX, respectively. The spiked homogenate was then left to equilibrate at 500 rpm for 3 hours before performing extractions at various times. While it is common practice to allow a minimum of 8 hours for drug equilibration in tissue to permit various binding equilibria to be established, in vivo lung sampling occurred as early as 10 min after drug administration during IVLP. As such, 3 hours was deemed a suitable compromise between common bioanalytical practice and the clinical conditions of the experiment. 15 g of lung homogenate was weighed for each time point of the extraction time profile, with 5 replicates investigated for each the two extraction phases (HLB and C_8_-SCX). Extraction was timed and performed under static conditions for all time points simultaneously to avoid any differences due to fluctuating binding equilibria as a function of time.

As shown in Fig. S8, extraction equilibrium was reached at 20 min for FOL and 30 min for F using HLB-coated fibers, and at 10 min for both compounds using C_8_-SCX. These results are in accordance with SPME fundamentals, which hold that longer equilibration times and higher amounts of extracted analyte are directly proportional to an increased affinity between the targeted compound and the coating. This explains why HLB coatings provide better method sensitivity, but also require equilibration times that are 2-3 times longer than those required for the C8-SCX coatings. Based on the results shown in Fig. S8, it is a safe assumption that the C18-SCX coating used for sample collection during IVLP and IV experiments would have achieved equilibrium between 10 and 30 min as a function of analyte affinity and coating performance. Therefore, it can be concluded that a 30-min extraction time was suitable for SPME sampling during pre-clinical IVLP and IV trials as it remains within the desired SPME equilibrium conditions.

5. Calibration curve and quality control

SPME fibers were used to perform in vivo sampling in the left lung during IVLP and IV trials. Study limitations (the release of external compounds (internal standard) was not permitted) inherent to this in vivo study conditions precluded the use of pre-loaded internal standards on the SPME fiber and as such, kinetic calibration methods could not be used for quantitation. Under the appropriate in vivo conditions, the amount of analyte extracted becomes independent of the sample weight or volume. Briefly, a bulk amount of lamb lung homogenate was spiked at the expected clinical FOLFOX concentration of 300 µg/g and then homogenized and agitated at 500 rpm for 1-2 hours. The bulk lung homogenate was then portioned at weights of 1, 5, 10, and 15 g, with extractions being performed simultaneously from each weight in replicates of 5 to avoid any errors associated with time delay or binding equilibria. An extraction time of 30 min was employed. Fig. S2 (results for F shown, similar results obtained for FOL – not sown) shows that the extracted amount of F becomes independent at 10 g of lamb lung homogenate, which is consistent with SPME-related research for the quantification of doxorubicin from lung tissue.

To provide better ease of use for quantitation, the matrix-matched calibration curve was designed to correspond to an instrumental curve based on the amount extracted. This approach eliminates the need to continuously prepare and use lamb lung homogenate, which can be an arduous process. This method also reduces the use of the very high-concentration standards required for the appropriate weight of lung used, which proved difficult to achieve for some standards due to solubility limitations. Consequently, a matrix-matched external calibration curve was instead constructed in 15 g of lamb lung homogenate with a range of 2 µg/g-2000 µg/g (Fig. S3). Internal standards were then only added to the desorption solution, thus correcting for LC-MS injection, but not for extraction. This matrix-matched external calibration curve was made to correspond to an instrumental curve that ranged from 0.001 µg/mL to 1 µg/mL for F and 0.01 µg/mL to 3.5 µg/mL for FOL in order to reduce the use of large amounts of lung homogenate and to simplify the quantitation process. Ultimately, a limit of detection (LOD) of 25 µg/g and a limit of quantitation (LOQ) of 50 µg/g was achieved for both F and FOL. A quality control at concentrations of 100 µg/g and 500 µg/g were internally assessed via the back calculation of the validated matrix-matched lung homogenate calibration curve with these points excluded.

6. Sterilization and preconditioning

Clinical protocol requires the sterilization of all devices that will be used on animal models. While solvent sterilization was sufficient for these animal experiments, there are cases that may require more rigorous sterilization approaches. This type of preconditioning may affect the extraction efficiency of the fibers, which may in turn affect the various conditions that were evaluated. In this work, the following preconditioning solutions were tested and assessed: 50:50 methanol/water, which is the typical solution employed for SPME device conditioning; saline solution, which is often used in clinical settings; pure sterilized water; and no solution at all. These various solutions were tested for both autoclaved and non-autoclaved C_8_-SCX fibers since these fibers were the commercially available replacement for the C_18_-SCX fibers used to perform active sampling during the pre-clinical trials. During these tests, all fibers were preconditioned under their respective conditions for a minimum of 30 minutes, which was followed by a 30-minute static extraction in PBS solution spiked with 300 ug/mL of FOLFOX and metabolites, a 5 s manual rinse in water, and desorption in 50:50 acetonitrile/water for 60 minutes at 1500 rpm. Figures S4A and S4B reveal that the autoclaved and non-autoclaved fibers that were conditioned in 50:50 methanol/water performed significantly better than those that had been conditioned in saline, pure water, and no solution at all. Although saline appeared to outperform pure water and no-solution conditioning on average, there were no significant differences (P < 0.05) in the amounts of FOLFOX or metabolites extracted using the autoclaved and non-autoclaved devices, both within and between groups, that had been preconditioned using these solutions. However, there were significant differences (P < 0.05) in the amount of F and FOL extracted by the autoclaved and non-autoclaved fibers that had been conditioned with 50:50 methanol/water; specifically, non-autoclaved fibers extracted more of both compounds. This contrasts with previous results for the quantitation of doxorubicin in lung tissue, wherein autoclaved fibers out-performed non-autoclaved fibers. These results suggest that device pre-treatment and preconditioning have various effects that are compound-specific. Interestingly, the metabolite, 5,10-methyleneTHF, showed significantly (P < 0.05) improved recoveries under autoclaved conditions and preconditioning with 50:50 methanol/water compared to non-autoclaved conditions with the same preconditioning solvent. Conversely, no significant differences were observed for OX under the same conditions (Figure S4C). It is also important to mention that the autoclaved and non-autoclaved fibers were visibly different after being removed from the 50:50 methanol/water preconditioning solution. In contrast to the other solutions/conditions (saline, pure water, no preconditioning solution), which left the fibers with a white appearance, the 50:50 methanol/water solution left both the autoclaved and non-autoclaved fibers with a dark grey “hydrated” appearance.

7. Standard solutions, calibration curves, method validation and quality control

Folates are susceptible to degradation by oxidation and thus need to be protected with antioxidants. Dimethylsulfoxide (DMSO) has been documented as an antioxidant that is tissue compatible, miscible with LC-MS-compatible solvents, and is generally an effective cosolvent. Thus, in order to mimic the high FOLFOX concentrations that are administered in perfusate and expected to be observed in tissue during IVLP, DMSO was employed as a solvent to produce the highest resultant concentrations for FOL (100 mg/mL), OX (100 mg/mL), F (100 mg/mL), 5,10-methyleneTHF (10 mg/mL), 5-methylTHF (20 mg/mL), and THF (20 mg/mL). Conversely, water was used to produce the highest concentrations for 5FUrd (20 mg/mL), 5FdUrd (25 mg/mL), FBAL (10 mg/mL), FUPA (5 mg/mL), 5FdUMP (5 mg/mL), 5FdUTP (30 mg/mL), and FUTP (16 mg/mL). All standards were stored at –80C until further use. To reduce the number of freeze-thaw cycles these standards would be subjected to, each standard was diluted to working solutions of 100 µg/mL with water, which were then further diluted for method development and MS tuning. The limited availability (small amounts) and high costs of all metabolite standards, as well as their negligible recoveries via SPME extraction in matrix-deficient and especially matrix-abundant environments, precluded their quantitation in tissue in compliance with the in vivo SPME calibration method discussed in section 5 of in vivo SPME method development. However, it did allow for metabolite screening. A quality control (QC) at concentrations of 100 µg/g and 500 µg/g were internally assessed via the back calculations of the validated matrix-matched lung homogenate calibration curve with the respective points excluded prior to calculation. An accuracy and precision of 99.8% and 8% was achieved for the QC internally assessed at 100 µg/g while an accuracy and precision of 101% and 20% was achieved for the QC internally assessed at 500 µg/g.

A matrix-matched external calibration curve with no internal standard correction in lung was constructed for FOLFOX compounds such that 1 mL of standard mix at a specific concentration could be added to 15 g of lamb lung homogenate. This approach was appropriate, as internal standards were neither present nor could be used during in vivo sampling due to clinical limitations imposed by the hospital as a safety precaution for the animals. Thus, 1.5 mL of FOLFOX stock standards diluted with water were made at the following concentrations: 30, 75, 150, 375, 750, 1,125, 1,500, 3,750, 7,500, 15,000 and 30,000 µg/mL; in order to produce an external lung homogenate calibration curve corresponding to concentrations of 2, 5, 10, 25, 50, 75, 100, 250, 500, 1,000, and 2,000 µg/g. Please note however, that internal standards were added to a fixed volume of desorption solution after the desorption step and LC-HRMS analysis. In accordance with the FDA’s guidelines for bioanalytical method validation, the LOD and LOQ were found to be 25 µg/g and 50 µg/g, respectively, for both FOL and F, with an achieved linearity (R^2^) of 0.99 for both compounds (Fig. S3A and S3C).

To provide better ease of use for quantitation, the matrix-matched calibration curve was designed to correspond to an instrumental curve based on the amount extracted. This approach eliminates the need to continuously prepare and use lamb lung homogenate, which can be an arduous process. This method also reduces the use of the very high-concentration standards required for the appropriate weight of lung used, which proved difficult to achieve for some standards due to solubility limitations. Fig. S3B and S6D show the absolute amounts (ng) of FOL and F that were extracted at each point of the matrix-matched external lung calibration curve. While this method of quantification allows for a more efficient and sustainable workflow, it is worth mentioning that it is only useful for 1.5 cm length C_8_-SCX coated fibers with a thickness of 40 µm. Other SPME devices using a different coating and/or dimensions of the coating will likely produce different results and need to be thus optimized for. Furthermore, the instrumental calibration curve should be constructed in the same way each time, to avoid issues with accuracy and precision. As such, the instrumental curve used herein was constructed via serial dilutions of a 40 µg/mL stock solution such that 10 µL of each instrumental stock from 10 ng/mL to 40 µg/mL could be diluted to 100 µL, thereby producing an instrumental curve that ranged from 1 ng/mL to 4 µg/mL. The obtained LODs and LOQs corresponded to an extracted amount of 90 ng and 295 ng for FOL, and 12.5 ng and 30 ng for F. The instrumental curve was used to determine the absolute amounts of FOL and F obtained in clinical IVLP and IV samples that were below the LOQs of the lung calibration curve. In addition, the absolute amounts of FOL and F were also determined for perfusate samples instead of the appropriate concentration. This drawback was due to the restricted availability of Steen^TM^ solution to persons strictly within clinical settings. These restrictions were a result of very high cost of this commodity (approx. $2000-3000 CAD per 500 mL) and its exclusive use for organ preservation. As a result, an external matrix matched calibration curve could not be constructed. The instrumental curves for FOL and F correlated to the matrix-matched external lamb lung homogenate calibration curve, were used for absolute quantitation of low concentration lung samples and perfusate samples. Both instrumental curves produced a linearity (R^2^) of 0.999 each, with an LOD and LOQ of 1 ng/mL and 5 ng/mL for FOL, and an LOD and LOQ of 5 ng/mL each for F.

**Tables**

**Table S1.** Liquid chromatography gradient conditions used with the Sequant® Zic®-pHILIC column (100 x 2.1 mm, 5µm particle size) for (OX), F, and F metabolites.

| **Time (min)**  **Total flow = 300 µL/min** | **% Mobile phase A: water + 0.1 % acetic acid + 5mM ammonium acetate** | **% Mobile phase B: acetonitrile + 0.1 % acetic acid + 5mM ammonium acetate** |
| --- | --- | --- |
| 0.00 | 10 | 90 |
| 1.00 | 10 | 90 |
| 4.00 | 70 | 30 |
| 4.70 | 70 | 30 |
| 4.90 | 10 | 90 |
| 8.00 | 10 | 90 |

**Table S2**. Mass spectrometry conditions and observed chromatographic retention times for OX, F, and F metabolites. Negative mode ionization was employed.

| **Compound** | **Parent mass** | **Product mass** | **S-LENS** | **Collision energy** | **Retention time (min)** |
| --- | --- | --- | --- | --- | --- |
| FBAL | 106 | 86 | 46 | 11 | 5.66 |
| FBAL-IS | 109 | 89 | 47 | 11 | 5.66 |
| 5-fluorouracil | 129 | 42 | 49 | 19 | 1.26 |
| 5-chlorouracil | 145 | 42 | 52 | 20 | 1.21 |
| FUPA | 149 | 86 | 41 | 18 | 5.51 |
|  |  | 106 * |  | 12 |  |
| FUPA-IS | 131 | 88 | 38 | 12 | 5.30 |
| 5-FdUrd | 245 | 155 | 69 | 17 | 1.09 |
| 5-FdUrd-IS | 248 | 132 | 69 | 16 | 1.11 |
|  |  | 158 * |  | 17 |  |
| 5-FUrd | 261 | 129 | 72 | 10 | 1.55 |
|  |  | 171* |  | 16 |  |
| 5-FUrd-IS | 264 | 132* | 72 | 17 | 1.57 |
|  |  | 171 |  | 16 |  |
| 5-FdUMP | 325 | 79 | 82 | 53 | 5.54 |
|  |  | 129 * |  | 23 |  |
| UMP-IS | 325 | 97 | 105 | 24 | 5.74 |
|  |  | 113 * |  | 28 |  |
| 5-FUTP | 501 | 159 * | 112 | 39 | 6.63 |
|  |  | 403 |  | 20 |  |
| 5-FdUTP | 485 | 159 | 104 | 35 | 6.56 |
|  |  | 257 |  | 27 |  |
|  |  | 387 * |  | 20 |  |
| Oxaliplatin | 396 | 89 | 95 | 19 | 4.60 |
|  |  | 196 * |  | 37 |  |
|  |  | 284 |  | 21 |  |
| Carboplatin | 370 | 352 | 133 | 12 | 5.22 |

Some compounds had more than one transition. Transitions which are denoted by (*) were used as the quantifier ion. The other transition(s) were used as qualifier ions. IS means internal standard.

The Ion Max heated electrospray ionization source was run at 2 kV at position D with a vaporizer and capillary temperature of 275 °C, and a sheath gas, ion sweep gas, and auxiliary gas of 35, 0, and 5, respectively.

**Table S3**. Liquid chromatography gradient conditions for the Discovery HS F5-3 (PFP) column (100 x 2.1 mm, 5µm particle size) used for FOL and its metabolites, 5,10-methyleneTHF, 5-methylTHF, THF.

| **Time (min)**  **Total flow = 300 µL/min** | **Mobile phase A: water + 0.25% acetic acid + 0.05% formic acid** | **Mobile phase B: acetonitrile + 0.25% acetic acid + 0.05% formic acid** |
| --- | --- | --- |
| 0 | 95 | 5 |
| 0.33 | 95 | 5 |
| 0.83 | 85 | 15 |
| 2.17 | 76 | 24 |
| 2.50 | 50 | 50 |
| 2.67 | 10 | 90 |
| 4.20 | 10 | 90 |
| 7.00 | 95 | 5 |
| 8.50 | 95 | 5 |

**Table S4**. Mass spectrometry conditions and observed chromatographic retention times for FOL and its metabolites, 5,10-methyleneTHF, 5-methylTHF, THF. Positive ionization was employed.

| **Compound** | **Parent mass** | **Product mass** | **S-LENS** | **Collision energy** | **Retention time** |
| --- | --- | --- | --- | --- | --- |
| Folinic acid | 474 | 327 | 109 | 18 | 1.33 |
| 5,10-methylTHF | 458 | 311 | 79 | 20 | 5.00 |
| 5-methylTHF | 460 | 180 | 56 | 39 | 5.46 |
|  |  | 313 * |  | 19 |  |
| THF | 446 | 299 | 113 | 19 | 5.14 |
| Folic acid-d2 | 444 | 297 | 95 | 17 | 1.35 |

The Ion Max heated electrospray ionization source was run at 1.3 kV at position B with a vaporizer and capillary temperature of 275 °C, and a sheath gas, ion sweep gas, and auxiliary gas of 30, 2, and 30, respectively.

**Table S5:** High- and medium confidence annotated features changing over the course of IVLP (Lung blank until the end of reperfusion IVLP T5) with FOLFOX administration

|  | **Lung Blank** | **IVLP L0** | **IVLP L1** | **IVLP T0** | **IVLP T1** | **IVLP T2** | **IVLP T3** | **IVLP T4** | **IVLP T5** |
| --- | --- | --- | --- | --- | --- | --- | --- | --- | --- |
| **amino acids** | L-Glutamine | L-Tryptophan | Lipoyllysine | Beta-Citryl-L-glutamic acid | L-Asparagine | Beta-Citryl-L-glutamic acid | Lipoyllysine | Beta-Citryl-L-glutamic acid | Beta-Citryl-L-glutamic acid |
|  | L-Phenylalanine | L-Phenylalanine | D-Tryptophan | Lipoyllysine | L-Threonine | Lipoyllysine | N(6)-(Octanoyl)lysine | Lipoyllysine | Lipoyllysine |
|  | L-Tyrosine | Lipoyllysine | L-Tryptophan | N-acetyltryptophan | Beta-Citryl-L-glutamic acid | L-Tryptophan | N-Acetylglutamine | L-Tryptophan | L-Tryptophan |
|  | D-Serine | N-Acetyl-L-methionine | Kynurenic acid | D-Tryptophan | Lipoyllysine | Kynurenic acid | D-Lysine | L-Asparagine | Kynurenic acid |
|  | N6,N6,N6-Trimethyl-L-lysine | L-Aspartic acid | L-Phenylalanine | (E)-2-Methylglutaconic acid | L-Aspartic acid | L-Phenylalanine | Kynurenic acid | L-Threonine | Tridecanoylglycine |
|  | Lipoyllysine | L-Serine | N-Acetylhistamine | L-Tryptophan | N-acetyltryptophan | N-Acetylhistamine | L-Lysine | 2-Hydroxyglutarate | N-Undecanoylglycine |
|  | L-Methionine | L-Lysine | Tridecanoylglycine | L-Phenylalanine | N-Acetylglutamine | N-Undecanoylglycine | L-Histidine | N-acetyltryptophan | Myristoylglycine |
|  | Homocitrulline | L-Histidine | N-Undecanoylglycine | N-Acetylhistamine | N-Acetylglutamic acid | Myristoylglycine | L-Phenylalanine | D-Glutamine | Palmitoylglycine |
|  | L-Cystine | L-Asparagine | N-Nonanoylglycine | N-Undecanoylglycine | Kynurenic acid | Palmitoylglycine | N-Acetylhistamine | L-Phenylalanine |  |
|  | L-Lysine | L-Threonine | N-Lauroylglycine | Tridecanoylglycine | 3-Hydroxymethylglutaric acid | Tridecanoylglycine | Tridecanoylglycine | N-Acetylhistamine |  |
|  | L-Asparagine | o-Tyrosine | N-Decanoylglycine | N-Nonanoylglycine | L-Phenylalanine | N-Nonanoylglycine | N-Undecanoylglycine | Tridecanoylglycine |  |
|  | L-Threonine | D-Glutamine |  | N-Decanoylglycine | N-Acetylhistamine | N-Decanoylglycine | N-Nonanoylglycine | N-Undecanoylglycine |  |
|  | L-Proline | N-Acetylglutamic acid |  | Myristoylglycine | Pristanoylglycine |  | N-Lauroylglycine | Palmitoylglycine |  |
|  | L-Glutamic acid | Kynurenic acid |  |  | Myristoylglycine |  | N-Decanoylglycine | N-Nonanoylglycine |  |
|  | N-Acetylglutamic acid | L-2-Hydroxyglutaric acid |  |  | Palmitoylglycine |  |  | N-Lauroylglycine |  |
|  | Kynurenic acid | L-Glutamine |  |  | Tridecanoylglycine |  |  | N-Decanoylglycine |  |
|  | L-Leucine | D-2-Hydroxyglutaric acid |  |  | N-Undecanoylglycine |  |  | Dimethylglycine |  |
|  | L-Isoleucine | 3-Hydroxyglutaric acid |  |  | N-Nonanoylglycine |  |  |  |  |
|  | N-Acetyl-L-methionine | L-Tyrosine |  |  | N-Decanoylglycine |  |  |  |  |
|  | L-Arginine | N-Acetylhistamine |  |  |  |  |  |  |  |
|  | L-Histidine | o-Tyrosine |  |  |  |  |  |  |  |
|  | L-Tryptophan | Beta-Tyrosine |  |  |  |  |  |  |  |
|  | Ne,Ne dimethyllysine | L-Tyrosine |  |  |  |  |  |  |  |
|  | Tridecanoylglycine | N-Undecanoylglycine |  |  |  |  |  |  |  |
|  | Pristanoylglycine | Tridecanoylglycine |  |  |  |  |  |  |  |
|  | N-Undecanoylglycine | N-Nonanoylglycine |  |  |  |  |  |  |  |
|  | Guanidoacetic acid | N-Lauroylglycine |  |  |  |  |  |  |  |
|  | 3-Hydroxymethylglutaric acid | N-Decanoylglycine |  |  |  |  |  |  |  |
|  | Pyroglutamic acid | Beta-Tyrosine |  |  |  |  |  |  |  |
|  |  | Dimethylglycine |  |  |  |  |  |  |  |
| **peptides** | Glu-Val | Phenylalanyl-Lysine | L-gamma-glutamyl-L-valine | Glutamylvaline | Tyrosyl-Glutamate | L-gamma-glutamyl-L-valine | Glutamylvaline | Glutamylvaline | Leucyl-Aspartate |
|  | L-gamma-glutamyl-L-valine | Lysyl-Phenylalanine | L-beta-aspartyl-L-leucine | L-gamma-glutamyl-L-valine | Leucyl-Aspartate | L-beta-aspartyl-L-phenylalanine | Prolyl-Asparagine | Tyrosyl-Glutamate | Isoleucyl-Aspartate |
|  | L-beta-aspartyl-L-leucine | Alanyl-Glycine | Arginyl-Arginine | L-beta-aspartyl-L-leucine | Isoleucyl-Aspartate | L-beta-aspartyl-L-leucine | Phenylalanyl-Aspartate | Leucyl-Aspartate | Histidinyl-Histidine |
|  | Cysteineglutathione disulfide | L-alpha-glutamyl-L-hydroxyproline |  | L-alpha-glutamyl-L-hydroxyproline | Glycyl-Hydroxyproline | L-Aspartyl-L-phenylalanine | Leucyl-Aspartate | Isoleucyl-Aspartate | Hydroxyprolyl-Cysteine |
|  | L-Cysteinylglycine disulfide | Alanylglycine |  |  | Glutamyl-Tyrosine | Arginyl-Arginine | Isoleucyl-Aspartate | Glutamyl-Tyrosine | Cysteinyl-Hydroxyproline |
|  | Glutarylglycine | L-Threo-3-Phenylserine |  |  | Aspartyl-Leucine |  | Histidinyl-Histidine | Aspartyl-Leucine | Aspartyl-Leucine |
|  |  | Glutarylglycine |  |  | Aspartyl-Isoleucine |  | Glycyl-Hydroxyproline | Aspartyl-Isoleucine | Aspartyl-Isoleucine |
|  |  | Arginyl-Arginine |  |  | L-glycyl-L-hydroxyproline |  | Aspartyl-Phenylalanine | Alanyl-Glycine | L-gamma-glutamyl-L-valine |
|  |  |  |  |  | L-gamma-glutamyl-L-valine |  | Aspartyl-Leucine | L-gamma-glutamyl-L-valine | L-beta-aspartyl-L-leucine |
|  |  |  |  |  | L-beta-aspartyl-L-leucine |  | Aspartyl-Isoleucine | L-beta-aspartyl-L-leucine |  |
|  |  |  |  |  | Glutarylglycine |  | Asparaginyl-Proline | Alanylglycine |  |
|  |  |  |  |  | Arginyl-Arginine |  | L-glycyl-L-hydroxyproline | Oxidized glutathione |  |
|  |  |  |  |  |  |  | L-gamma-glutamyl-L-valine |  |  |
|  |  |  |  |  |  |  | L-beta-aspartyl-L-phenylalanine |  |  |
|  |  |  |  |  |  |  | L-beta-aspartyl-L-leucine |  |  |
|  |  |  |  |  |  |  | L-alpha-glutamyl-L-hydroxyproline |  |  |
|  |  |  |  |  |  |  | L-Aspartyl-L-phenylalanine |  |  |
|  |  |  |  |  |  |  | Oxidized glutathione |  |  |
| **acylcarnitines** | 3-Dehydroxycarnitine | 3-Dehydroxycarnitine | 3-Dehydroxycarnitine | 3-Hydroxyhexadecadienoylcarnitine | 3-Hydroxyhexadecadienoylcarnitine | Hydroxybutyrylcarnitine | Arachidonyl carnitine | Hydroxybutyrylcarnitine | 3-Dehydroxycarnitine |
|  | L-Acetylcarnitine | Heptadecanoyl carnitine | 12-Hydroxy-12-octadecanoylcarnitine | 3-Hydroxy-9-hexadecenoylcarnitine | 3-Hydroxy-9-hexadecenoylcarnitine | 3-Hydroxyhexadecadienoylcarnitine | 3-Hydroxyhexadecadienoylcarnitine | Pimelylcarnitine | 3-Hydroxy-9-hexadecenoylcarnitine |
|  | 2-trans4-cis-Decadienoylcarnitine | Propionylcarnitine | Hydroxybutyrylcarnitine | 12-Hydroxy-12-octadecanoylcarnitine | Pimelylcarnitine | 3-Hydroxy-9-hexadecenoylcarnitine | Pimelylcarnitine | 2-trans4-cis-Decadienoylcarnitine | 2-trans4-cis-Decadienoylcarnitine |
|  | 6-Keto-decanoylcarnitine | L-Acetylcarnitine | Propionylcarnitine | Hydroxybutyrylcarnitine | 2-trans4-cis-Decadienoylcarnitine | Pimelylcarnitine | 12-Hydroxy-12-octadecanoylcarnitine | 12-Hydroxy-12-octadecanoylcarnitine | Hydroxybutyrylcarnitine |
|  | 12-Hydroxy-12-octadecanoylcarnitine |  |  | Propionylcarnitine | 12-Hydroxy-12-octadecanoylcarnitine | 12-Hydroxy-12-octadecanoylcarnitine | Hydroxybutyrylcarnitine | Propionylcarnitine | Propionylcarnitine |
|  | Hydroxyvalerylcarnitine |  |  | Arachidonyl carnitine | Hydroxybutyrylcarnitine | Propionylcarnitine | Propionylcarnitine | 3-Hydroxy-5 8-tetradecadiencarnitine | Malonylcarnitine |
|  | Propionylcarnitine |  |  |  | Hydroxypropionylcarnitine | Malonylcarnitine | Malonylcarnitine |  |  |
|  | L-Carnitine |  |  |  | Heptadecanoyl carnitine |  |  |  |  |
|  |  |  |  |  | Propionylcarnitine |  |  |  |  |
|  |  |  |  |  | Malonylcarnitine |  |  |  |  |
| **autacoids** |  |  |  |  | 7-hydroxy-D4-neuroprostane |  | 7-hydroxy-D4-neuroprostane | 7-hydroxy-D4-neuroprostane |  |
|  |  |  |  |  | 4-hydroxy-D4-neuroprostane |  | 4-hydroxy-D4-neuroprostane | 4-hydroxy-D4-neuroprostane |  |
|  |  |  |  |  | 20-hydroxy-E4-neuroprostane |  | 20-hydroxy-E4-neuroprostane | 20-hydroxy-E4-neuroprostane |  |
|  |  |  |  |  | 17-hydroxy-E4-neuroprostane |  | 19-Oxo-deoxycorticosterone | 17-hydroxy-E4-neuroprostane |  |
|  |  |  |  |  | 14-hydroxy-E4-neuroprostane |  | 17-hydroxy-E4-neuroprostane | 14-hydroxy-E4-neuroprostane |  |
|  |  |  |  |  | Resolvin D1 |  | 14-hydroxy-E4-neuroprostane | Resolvin D1 |  |
|  |  |  |  |  | Resolvin D2 |  | Resolvin D1 | Resolvin D2 |  |
|  |  |  |  |  |  |  | Resolvin D2 |  |  |
| **eicosanoids** | Omega-Carboxy-trinor-leukotriene B4 | 18-Carboxy-dinor- leukotriene E4 | Omega-Carboxy-trinor-leukotriene B4 | Leukotriene B4 ethanolamide | Omega-Carboxy-trinor-leukotriene B4 | Leukotriene B4 ethanolamide | Omega-Carboxy-trinor-leukotriene B4 | 18-Carboxy-dinor- leukotriene E4 | Omega-Carboxy-trinor-leukotriene B4 |
|  | 18-Carboxy-dinor- leukotriene E4 | 13,14-Dihydro Prostaglandin F-1a | 13,14-Dihydro Prostaglandin F-1a | 5-Oxo-6-trans-leukotriene B4 | 18-Carboxy-dinor- leukotriene E4 | 67-dihydro-5-oxo-12-epi- leukotriene B4 | 18-Carboxy-dinor- leukotriene E4 | 6,7-dihydro-5-oxo-12-epi- leukotriene B4 | 18-Carboxy-dinor- leukotriene E4 |
|  | Leukotriene B4 ethanolamide | 12,13-DHOME | 12,13-DHOME | Leukotriene B5 | Leukotriene B4 ethanolamide | 1011-dihydro-12-oxo- leukotriene B4 | Leukotriene B4 ethanolamide | 10,11-dihydro-12-oxo- leukotriene B4 | 6,7-dihydro-5-oxo-12-epi- leukotriene B4 |
|  | Leukotriene B5 | 9,10-DHOME | 9,10-DHOME | 12-Keto-leukotriene B4 | Prostaglandin F2a ethanolamide | 12(S)-Leukotriene B4 | Leukotriene B5 | 12(S)-Leukotriene B4 | 10,11-dihydro-12-oxo- leukotriene B4 |
|  | 12-Keto-leukotriene B4 |  |  | Prostaglandin F2a ethanolamide | 15-Deoxy-d-1214- Prostaglandin J2 | 6-trans-12-epi-Leukotriene B4 | 12-Keto-leukotriene B4 | 6-trans-12-epi-Leukotriene B4 | 12(S)-Leukotriene B4 |
|  | 10,11-dihydro-20-dihydroxy- leukotriene B4 |  |  | Prostaglandin-c2 | 1314-Dihydro Prostaglandin F-1a | 6-trans-Leukotriene B4 | Prostaglandin F2a ethanolamide | 6-trans-Leukotriene B4 | 6-trans-12-epi-Leukotriene B4 |
|  | Prostaglandin E2 ethanolamide |  |  | bicyclo- Prostaglandin E2 |  | Leukotriene B4 | 13,14-Dihydro Prostaglandin F-1a | Leukotriene B4 | 6-trans-Leukotriene B4 |
|  | 13,14-Dihydro Prostaglandin F-1a |  |  | 15d Prostaglandin D2 |  | Prostaglandin F2a ethanolamide | Prostaglandin-c2 | Omega-Carboxy-trinor-leukotriene B4 | Leukotriene B4 |
|  | Prostaglandin-c2 |  |  | 15-Deoxy-d-12,14- Prostaglandin J2 |  | 1314-Dihydro Prostaglandin F-1a | bicyclo- Prostaglandin E2 | 1314-Dihydro Prostaglandin F-1a | 5-Oxo-6-trans-leukotriene B4 |
|  | bicyclo- Prostaglandin E2 |  |  | Delta-12-Prostaglandin J2 |  | Prostaglandin C1 | 15d Prostaglandin D2 | Prostaglandin C1 | Leukotriene B5 |
|  | 15d Prostaglandin D2 |  |  | Prostaglandin B2 |  | 9-Deoxy-delta12- Prostaglandin D2 | Delta-12-Prostaglandin J2 | 9-Deoxy-delta12- Prostaglandin D2 | 12-Keto-leukotriene B4 |
|  | Delta-12-Prostaglandin J2 |  |  | Prostaglandin A2 |  | 15-Deoxy-d-12,14-PGJ2 | Prostaglandin B2 | Prostaglandin B1 | Prostaglandin C1 |
|  | Prostaglandin B2 |  |  | Prostaglandin J2 |  | Prostaglandin B1 | Prostaglandin A2 | Prostaglandin A1 | 9-Deoxy-delta12- Prostaglandin D2 |
|  | Prostaglandin A2 |  |  | 15-Keto-13,14-dihydroprostaglandin A2 |  | Prostaglandin A1 | Prostaglandin J2 | 8-iso- Prostaglandin A1 | 15-Deoxy-d-12,14- Prostaglandin J2 |
|  | Prostaglandin J2 |  |  | 12,13-DHOME |  | 8-iso- Prostaglandin A1 | 15-Keto-1314-dihydroprostaglandin A2 | Prostaglandin F2a ethanolamide | Prostaglandin B1 |
|  | 15-Keto-13,14-dihydroprostaglandin A2 |  |  | 9,10-DHOME |  | 13,14-Dihydro Prostaglandin F-1a | 13,14-Dihydro Prostaglandin F-1a | 12,20-DiHETE | Prostaglandin A1 |
|  | Prostaglandin G1 |  |  |  |  | 12,20-DiHETE | 12,13-DHOME | 5-HPETE | 8-iso- Prostaglandin A1 |
|  | 20-Hydroxy- Prostaglandin F2a |  |  |  |  | 17-HETE | 9,10-DHOME | 8,15-DiHETE | Prostaglandin F2a ethanolamide |
|  | 6-Keto-prostaglandin F1a |  |  |  |  | 13-HETE |  | 5,15-DiHETE | 13,14-Dihydro Prostaglandin F-1a |
|  | 13,14-Dihydro Prostaglandin F-1a |  |  |  |  | 10-HETE |  | 17,18-DiHETE | Prostaglandin-c2 |
|  | 17-HETE |  |  |  |  | 19(S)-HETE |  | 14,15-DiHETE | bicyclo- Prostaglandin E2 |
|  | 13-HETE |  |  |  |  | 5-HETE |  | 15H-11,12-EETA | 15d P Prostaglandin D2 |
|  | 10-HETE |  |  |  |  | 11,12-EpETrE |  | 12,13-DHOME | Delta-12-Prostaglandin J2 |
|  | 19(S)-HETE |  |  |  |  | 9-HETE |  | 9,10-DHOME | Prostaglandin B2 |
|  | 5-HETE |  |  |  |  | 8,15-DiHETE |  | 8(S)-HPETE | Prostaglandin A2 |
|  | 11,12-EpETrE |  |  |  |  | 5,15-DiHETE |  | 11(R)-HPETE | Prostaglandin J2 |
|  | 9-HETE |  |  |  |  | 17,18-DiHETE |  | 11H-14,15-EETA | 15-Keto-13,14-dihydroprostaglandin A2 |
|  | 12-HETE |  |  |  |  | 14,15-DiHETE |  | 12(R)-HPETE | 12,20-DiHETE |
|  | 11(R)-HETE |  |  |  |  | 12-HETE |  | 15(S)-HPETE | 5-HPETE |
|  | 16(R)-HETE |  |  |  |  | 15H-11,12-EETA |  | 12(S)-HPETE | 8,15-DiHETE |
|  | 8-HETE |  |  |  |  | 8(S)-HPETE |  |  | 5,15-DiHETE |
|  | 15(S)-HETE |  |  |  |  | 11(R)-HPETE |  |  | 17,18-DiHETE |
|  | 14R,15S-EpETrE |  |  |  |  | 11H-14,15-EETA |  |  | 14,15-DiHETE |
|  |  |  |  |  |  | 12(R)-HPETE |  |  | 15H-11,12-EETA |
|  |  |  |  |  |  | 11(R)-HETE |  |  | 12,13-DHOME |
|  |  |  |  |  |  | 16(R)-HETE |  |  | 9,10-DHOME |
|  |  |  |  |  |  | 15(S)-HPETE |  |  | 8(S)-HPETE |
|  |  |  |  |  |  | 12(S)-HPETE |  |  | 11(R)-HPETE |
|  |  |  |  |  |  | 15(S)-HETE |  |  | 11H-14,15-EETA |
|  |  |  |  |  |  | 14R,15S-EpETrE |  |  | 12(R)-HPETE |
|  |  |  |  |  |  | 9-HODE |  |  | 15(S)-HPETE |
|  |  |  |  |  |  | 9,10,13-TriHOME |  |  | 12(S)-HPETE |
|  |  |  |  |  |  | 12,13-EpOME |  |  | 9-HODE |
|  |  |  |  |  |  |  |  |  | 12,13-EpOME |
| **steriod hormones** |  |  |  | Cortisone | 5a-Dihydrotestosterone sulfate | 11beta20-Dihydroxy-3-oxopregn-4-en-21-oic acid | 19-Hydroxydeoxycorticosterone | 5a-Dihydrotestosterone sulfate | 11beta20-Dihydroxy-3-oxopregn-4-en-21-oic acid |
|  |  |  |  | Aldosterone | 11beta-Hydroxy-3,20-dioxopregn-4-en-21-oic acid |  | 11beta-Hydroxy-3,20-dioxopregn-4-en-21-oic acid | Androsterone sulfate |  |
|  |  |  |  | 11beta20-Dihydroxy-3-oxopregn-4-en-21-oic acid |  |  | 11beta20-Dihydroxy-3-oxopregn-4-en-21-oic acid | 11beta-Hydroxy-3,20-dioxopregn-4-en-21-oic acid |  |
|  |  |  |  |  |  |  | 21-Hydroxy-5b-pregnane-3,11,20-trione | 11beta20-Dihydroxy-3-oxopregn-4-en-21-oic acid |  |
|  |  |  |  |  |  |  | 21-Deoxycortisol |  |  |
|  |  |  |  |  |  |  | 11-Dehydrocorticosterone |  |  |
|  |  |  |  |  |  |  | Corticosterone |  |  |
|  |  |  |  |  |  |  | Cortexolone |  |  |
|  |  |  |  |  |  |  | Aldosterone |  |  |
| **purines, pyrimidines** | Thymidine 35-cyclic monophosphate | 7-Methylguanosine | Cytosine | Allopurinol riboside | Cytidine triphosphate | 5-Amino-6-ribitylamino uracil | Cytosine | 5-Methylthioadenosine | 5-Methylthioadenosine |
|  | 5-Methylthioadenosine | Cytosine | Deoxycytidine | 2-Deoxyinosine triphosphate | 5-Amino-6-ribitylamino uracil | Inosine triphosphate | Deoxycytidine | Cytosine | Cytosine |
|  | Cytosine | Deoxycytidine | Deoxyguanosine | Inosine | Thymidine 5-triphosphate |  | 2-Deoxyinosine triphosphate |  | Deoxycytidine |
|  | Deoxycytidine | Pseudouridine | Adenosine |  | Pseudouridine |  | 5-Aminoimidazole ribonucleotide |  | Inosine triphosphate |
|  | Pseudouridine | Uridine |  |  | Uridine |  |  |  |  |
|  | Uridine | Inosine triphosphate |  |  | 2-Deoxyinosine triphosphate |  |  |  |  |
|  | Adenosine phosphosulfate | Inosine |  |  | Inosine triphosphate |  |  |  |  |
|  | Adenosine monophosphate |  |  |  |  |  |  |  |  |
|  | Inosine triphosphate |  |  |  |  |  |  |  |  |
|  | Inosine |  |  |  |  |  |  |  |  |
| **other** | Oleamide | Oleamide | Oleamide | Arachidonoyl Serinol | Arachidonoyl Serinol | Arachidonoyl Serinol | Arachidonoyl Serinol | Arachidonoyl Serinol | S-Adenosylhomocysteine |
|  | NADH | Porphobilinogen | Porphobilinogen | Oleamide | Oleamide | Oleamide | Oleamide | Oleamide | Porphobilinogen |
|  | Fucose 1-phosphate | Ureidoisobutyric acid | Riboflavin | Porphobilinogen | Porphobilinogen | Porphobilinogen | Fucose 1-phosphate | Fucose 1-phosphate |  |
|  | S-Adenosylhomocysteine | dopamine sulfate | Taurine | Taurine |  |  | Porphobilinogen | Taurine |  |
|  | Homocysteine | Taurine |  |  |  |  | Taurine |  |  |
|  | Porphobilinogen | Creatinine |  |  |  |  |  |  |  |
|  | Norepinephrine sulfate |  |  |  |  |  |  |  |  |
|  | dopamine sulfate |  |  |  |  |  |  |  |  |
|  | FAD |  |  |  |  |  |  |  |  |
|  | Creatinine |  |  |  |  |  |  |  |  |
|  | Taurine |  |  |  |  |  |  |  |  |
|  | Creatine |  |  |  |  |  |  |  |  |

DHOME – dihydroxyoctadecenoic acid; HETE - hydroxyeicosatetraenoic acid; EpETrE (EETA)- epoxyeicosatrienoic acid; HPETE – hydroperoxyeicosanoic acid; HODE - hydroxyoctadecadienoic acid; NADH - reduced nicotinamide adenine dinucleotide; FDA - flavin adenine dinucleotide.

**Table S6**: High- and medium confidence annotated features from perfusate samples sampled on-site with SPME fibers in the hospital during IVLP with FOLFOX administration

|  | **T0** | **T1** | **T2** | **T3** |
| --- | --- | --- | --- | --- |
| **amino acids** | Beta-Citryl-L-glutamic acid | Beta-Citryl-L-glutamic acid | Beta-Citryl-L-glutamic acid | Beta-Citryl-L-glutamic acid |
|  | L-Tryptophan | N-acetyltryptophan | D-Tryptophan | N(6)-(Octanoyl)lysine |
|  | Kynurenic acid | N-Acetylhistamine | L-gamma-glutamyl-L-valine | N-acetyltryptophan |
|  | Lipoyllysine | L-Tryptophan | L-Tryptophan | D-Tryptophan |
|  | N-Acetylhistamine | L-Valine | L-Valine | N-Acetylglutamine |
|  | Tridecanoylglycine | Kynurenic acid | Kynurenic acid | L-Tryptophan |
|  | N-Decanoylglycine | Lipoyllysine | N-Acetylhistamine | Kynurenic acid |
|  | N-Undecanoylglycine | N-Undecanoylglycine | Lipoyllysine | L-Phenylalanine |
|  | N-Nonanoylglycine | Tridecanoylglycine | N-Undecanoylglycine | N-Acetylhistamine |
|  | Myristoylglycine | N-Decanoylglycine | N-Nonanoylglycine | Lipoyllysine |
|  | Palmitoylglycine | Palmitoylglycine | N-Decanoylglycine | N-Undecanoylglycine |
|  | Dimethylglycine | Pristanoylglycine | Myristoylglycine | Tridecanoylglycine |
|  |  | Myristoylglycine | Palmitoylglycine | N-Nonanoylglycine |
|  |  | Dimethylglycine | Methylglutaric acid | N-Decanoylglycine |
|  |  | Histidinal | L-2-Hydroxyglutaric acid | Pristanoylglycine |
|  |  |  | Histidinal | Palmitoylglycine |
|  |  |  |  | Methylglutaric acid |
|  |  |  |  | Histidinal |
| **peptides** | Histidinyl-Histidine | Gamma-glutamyl-Cysteine | Glutamylvaline | Glutamylvaline |
|  | Glutamylvaline | Prolyl-Asparagine | Gamma-glutamyl-Phenylalanine | Tyrosyl-Glutamate |
|  | Prolyl-Asparagine | Hydroxyprolyl-Cysteine | Tyrosyl-Glutamate | Tryptophyl-Arginine |
|  | Phenylalanyl-Lysine | Glycyl-Hydroxyproline | Tryptophyl-Arginine | Prolyl-Asparagine |
|  | Lysyl-Phenylalanine | Glutaminyl-Cysteine | Threoninyl-Arginine | Leucyl-Hydroxyproline |
|  | Leucyl-Aspartate | Cysteinyl-Gamma-glutamate | Prolyl-Asparagine | Leucyl-Aspartate |
|  | Isoleucyl-Aspartate | Cysteinyl-Hydroxyproline | Phenylalanyl-Gamma-glutamate | Isoleucyl-Hydroxyproline |
|  | Aspartyl-Leucine | Cysteinyl-Glutamine | Phenylalanyl-Glutamine | Isoleucyl-Aspartate |
|  | Aspartyl-Isoleucine | Asparaginyl-Proline | Phenylalanyl-Aspartate | Hydroxyprolyl-Leucine |
|  | Asparaginyl-Proline | Oxidized glutathione | Leucyl-Leucine | Hydroxyprolyl-Isoleucine |
|  | L-gamma-glutamyl-L-valine |  | Leucyl-Isoleucine | Hydroxyprolyl-Cysteine |
|  | L-beta-aspartyl-L-leucine |  | Leucyl-Hydroxyproline | Glycyl-Hydroxyproline |
|  | Arginyl-Arginine |  | Leucyl-Aspartate | Glutamyl-Tyrosine |
|  | Gamma-Glutamyltyrosine |  | Isoleucyl-Leucine | Cysteinyl-Hydroxyproline |
|  | Oxidized glutathione |  | Isoleucyl-Isoleucine | Aspartyl-Leucine |
|  |  |  | Isoleucyl-Hydroxyproline | Aspartyl-Isoleucine |
|  |  |  | Isoleucyl-Aspartate | Asparaginyl-Proline |
|  |  |  | Hydroxyprolyl-Isoleucine | Arginyl-Tryptophan |
|  |  |  | Hydroxyprolyl-Cysteine | L-glycyl-L-hydroxyproline |
|  |  |  | Glutamyl-Tyrosine | L-gamma-glutamyl-L-valine |
|  |  |  | Glutaminyl-Phenylalanine | L-beta-aspartyl-L-leucine |
|  |  |  | Cysteinyl-Hydroxyproline | Gamma-Glutamyltyrosine |
|  |  |  | Aspartyl-Phenylalanine | Arginyl-Arginine |
|  |  |  | Aspartyl-Leucine | Oxidized glutathione |
|  |  |  | Aspartyl-Isoleucine |  |
|  |  |  | Asparaginyl-Proline |  |
|  |  |  | Arginyl-Tryptophan |  |
|  |  |  | Arginyl-Threonine |  |
|  |  |  | L-beta-aspartyl-L-phenylalanine |  |
|  |  |  | L-beta-aspartyl-L-leucine |  |
|  |  |  | L-alpha-glutamyl-L-hydroxyproline |  |
|  |  |  | L-Aspartyl-L-phenylalanine |  |
| **acylcarnitines** | Pimelylcarnitine | Pimelylcarnitine | 3-Hydroxyhexadecadienoylcarnitine | Pimelylcarnitine |
|  | 2-trans4-cis-Decadienoylcarnitine | 3-Hydroxyhexadecadienoylcarnitine | Pimelylcarnitine | Hydroxybutyrylcarnitine |
|  | 12-Hydroxy-12-octadecanoylcarnitine | 3-Hydroxy-9-hexadecenoylcarnitine | trans-2-Dodecenoylcarnitine | Arachidonyl carnitine |
|  | Hydroxybutyrylcarnitine | 3-Hydroxy-5 8-tetradecadiencarnitine | 12-Hydroxy-12-octadecanoylcarnitine | 6-Keto-decanoylcarnitine |
|  | Propionylcarnitine | 2-trans4-cis-Decadienoylcarnitine | Hydroxybutyrylcarnitine | 12-Hydroxy-12-octadecanoylcarnitine |
|  | Malonylcarnitine | 12-Hydroxy-12-octadecanoylcarnitine | Hydroxypropionylcarnitine | Propionylcarnitine |
|  | Hydroxypropionylcarnitine | Hydroxybutyrylcarnitine | Propionylcarnitine | Arachidonyl carnitine |
|  |  | Propionylcarnitine | Malonylcarnitine | 2,6 Dimethylheptanoyl carnitine |
|  |  | Malonylcarnitine | 3-Hydroxy-9-hexadecenoylcarnitine | Malonylcarnitine |
| **autacoids** | 7-hydroxy-D4-neuroprostane | 7-hydroxy-D4-neuroprostane | 7-hydroxy-D4-neuroprostane | 7-hydroxy-D4-neuroprostane |
|  | 4-hydroxy-D4-neuroprostane | 4-hydroxy-D4-neuroprostane | 4-hydroxy-D4-neuroprostane | 4-hydroxy-D4-neuroprostane |
|  | 20-hydroxy-E4-neuroprostane | 20-hydroxy-E4-neuroprostane | 20-hydroxy-E4-neuroprostane | 20-hydroxy-E4-neuroprostane |
|  | 17-hydroxy-E4-neuroprostane | 17-hydroxy-E4-neuroprostane | 17-hydroxy-E4-neuroprostane | 17-hydroxy-E4-neuroprostane |
|  | 14-hydroxy-E4-neuroprostane | 14-hydroxy-E4-neuroprostane | 14-hydroxy-E4-neuroprostane | 14-hydroxy-E4-neuroprostane |
|  | Resolvin D1 | Resolvin D1 | Resolvin D1 | Resolvin D1 |
|  | Resolvin D2 | Resolvin D2 | Resolvin D2 | Resolvin D2 |
|  |  |  |  | Resolvin D5 |
|  |  |  |  | Neuroprotectin D1 |
| **eicosanoids** | Omega-Carboxy-trinor-leukotriene B4 | Omega-Carboxy-trinor-leukotriene B4 | Omega-Carboxy-trinor-leukotriene B4 | Omega-Carboxy-trinor-leukotriene B4 |
|  | 18-Carboxy-dinor- leukotriene E4 | 18-Carboxy-dinor- leukotriene E4 | 18-Carboxy-dinor- leukotriene E4 | 18-Carboxy-dinor- leukotriene E4 |
|  | 6,7-dihydro-5-oxo-12-epi- leukotriene B4 | 13E-Tetranor-16-carboxy- leukotriene E4 | Leukotriene B4 ethanolamide | Leukotriene B4 ethanolamide |
|  | 6,7-dihydro-12-epi- leukotriene B4 | Leukotriene B4 ethanolamide | 6,7-dihydro-12-epi- leukotriene B4 | 5-Oxo-6-trans-leukotriene B4 |
|  | 12-oxo-20-dihydroxy-leukotriene B4 | Prostaglandin F2a ethanolamide | 5-Oxo-6-trans-leukotriene B4 | Leukotriene B5 |
|  | 12-oxo-10,11-dihydro-20-COOH- leukotriene B4 | 1314-Dihydro PGF-1a | 10,11-dihydro-leukotriene B4 | 12-Keto-leukotriene B4 |
|  | 10,11-dihydro-leukotriene B4 | 12(13)Ep-9-KODE | Leukotriene B5 | 13,14-Dihydro Prostaglandin F-1a |
|  | 10,11-dihydro-12-oxo- leukotriene B4 | 9,10,13-TriHOME | 12-Keto-leukotriene B4 | Prostaglandin F2a ethanolamide |
|  | 20-Carboxy-leukotriene B4 | 9,12,13-TriHOME | 12-Keto-tetrahydro-leukotriene B4 | 5,6-Dihydroxyprostaglandin F1a |
|  | 12(S)-Leukotriene B4 | 12,13-DHOME | Prostaglandin F2a ethanolamide | Prostaglandin-c2 |
|  | 6-trans-12-epi-Leukotriene B4 | 9,10-DHOME | 13,14-Dihydro Prostaglandin F-1a | bicyclo- Prostaglandin E2 |
|  | 6-trans-Leukotriene B4 |  | Prostaglandin-c2 | 15d Prostaglandin D2 |
|  | 12-Keto-tetrahydro-leukotriene B4 |  | bicyclo- Prostaglandin E2 | Delta-12-Prostaglandin J2 |
|  | Leukotriene B4 |  | 15d Prostaglandin D2 | Prostaglandin B2 |
|  | Prostaglandin F2a ethanolamide |  | Delta-12-Prostaglandin J2 | Prostaglandin A2 |
|  | 13,14-Dihydro Prostaglandin F-1a |  | Prostaglandin B2 | Prostaglandin J2 |
|  | Prostaglandin C1 |  | Prostaglandin A2 | 15-Keto-1314-dihydroprostaglandin A2 |
|  | 9-Deoxy-delta12- Prostaglandin D2 |  | Prostaglandin J2 | 13,14-Dihydro Prostaglandin F-1a |
|  | Prostaglandin B1 |  | 15-Keto-13,14-dihydroprostaglandin A2 | 12(13)Ep-9-KODE |
|  | Prostaglandin A1 |  | 13,14-Dihydro Prostaglandin F-1a | 17-HETE |
|  | 8-iso- Prostaglandin A1 |  | 17-HETE | 13-HETE |
|  | 13,14-Dihydro Prostaglandin F-1a |  | 13-HETE | 10-HETE |
|  | 12,20-DiHETE |  | 10-HETE | 19(S)-HETE |
|  | 5-HPETE |  | 19(S)-HETE | 5-HETE |
|  | 8,15-DiHETE |  | 5-HETE | 11,12-EpETrE |
|  | 5,15-DiHETE |  | 11,12-EpETrE | 9-HETE |
|  | 17,18-DiHETE |  | 9-HETE | 12-HETE |
|  | 14,15-DiHETE |  | 12-HETE | 12,13-DHOME |
|  | 15H-11,12-EETA |  | 12,13-DHOME | 9,10-DHOME |
|  | 12,13-DHOME |  | 9,10-DHOME | 11(R)-HETE |
|  | 9,10-DHOME |  | 11(R)-HETE | 16(R)-HETE |
|  | 8(S)-HPETE |  | 16(R)-HETE | 8-HETE |
|  | 11(R)-HPETE |  | 8-HETE | 15(S)-HETE |
|  | 11H-14,15-EETA |  | 15(S)-HETE | 14R,15S-EpETrE |
|  | 12(R)-HPETE |  | 5,6-DHET | 9,10,13-TriHOME |
|  | 15(S)-HPETE |  | 11,12-DiHETrE | 9,12,13-TriHOME |
|  | 12(S)-HPETE |  | 89-DiHETrE |  |
|  | 5,6-DHET |  | 14R,15S-EpETrE |  |
|  | 11,12-DiHETrE |  | 14,15-DiHETrE |  |
|  | 8,9-DiHETrE |  | 9,10,13-TriHOME |  |
|  | 14,15-DiHETrE |  | 9,12,13-TriHOME |  |
|  | 9-HODE |  |  |  |
|  | 12,13-EpOME |  |  |  |
| **steriod hormones** | Aldosterone | 11beta-Hydroxy-3,20-dioxopregn-4-en-21-oic acid | 19-Oxo-deoxycorticosterone | Aldosterone |
|  | 11beta-Hydroxy-3,20-dioxopregn-4-en-21-oic acid | 11beta20-Dihydroxy-3-oxopregn-4-en-21-oic acid | 11beta-Hydroxy-3,20-dioxopregn-4-en-21-oic acid | 19-Oic-deoxycorticosterone |
|  | 11-Dehydrocorticosterone |  |  | 11beta-Hydroxy-3,20-dioxopregn-4-en-21-oic acid |
|  | Cortisone |  |  | 11beta20-Dihydroxy-3-oxopregn-4-en-21-oic acid |
| **purines, pyrimidines** | Inosine triphosphate | 5-Amino-6-ribitylamino uracil | Inosine triphosphate | 7-Methylguanosine |
|  |  | 2-Deoxyinosine triphosphate | Cytosine | 5-Amino-6-ribitylamino uracil |
|  |  | 7-Methylguanosine | 2-Deoxyinosine triphosphate | 2-Deoxyinosine triphosphate |
|  |  | Inosine triphosphate | Pseudouridine | Cytosine |
|  |  | Pseudouridine | Uridine | Inosine triphosphate |
|  |  | Uridine |  | 5-Fluorouridine |
| **other** | Arachidonoyl Serinol | Arachidonoyl Serinol | Arachidonoyl Serinol | L-Urobilinogen |
|  | Oleamide | Oleamide | Oleamide | Arachidonoyl Serinol |
|  | Porphobilinogen | Fucose 1-phosphate | Norepinephrine sulfate | Ubiquinol-10 |
|  | Riboflavin | 5-Aminoimidazole ribonucleotide |  | Oleamide |
|  |  | Porphobilinogen |  | 5-Aminoimidazole ribonucleotide |

KODE - oxooctadecenoic acid; DHOME – dihydroxyoctadecenoic acid; HETE - hydroxyeicosatetraenoic acid; EpETrE (EETA)- epoxyeicosatrienoic acid; HPETE – hydroperoxyeicosanoic acid; HODE - hydroxyoctadecadienoic acid.

**Table S7**: High- and medium confidence annotated features from lungs sampled *in vivo* with SPME fibers in the hospital during IV administration with FOLFOX.

|  | **IV_Blank** | **IV_TL** | **IV_T0** | **IV_T1** | **IV_T2** | **IV_T3** |
| --- | --- | --- | --- | --- | --- | --- |
| **amino acids** | L-Tryptophan | L-Tryptophan | N-Acetyl-L-methionine | L-Tryptophan | Beta-Citryl-L-glutamic acid | D-Serine |
|  | L-Glutamine | L-Phenylalanine | N6,N6,N6-Trimethyl-L-lysine | L-Glutamine | N6,N6,N6-Trimethyl-L-lysine | L-Glutamine |
|  | L-Arginine | Beta-Citryl-L-glutamic acid | L-Methionine | L-Tyrosine | L-Methionine | L-Proline |
|  | L-Lysine | N-Acetyl-L-methionine | L-Arginine | N-Acetyl-L-methionine | L-Arginine | L-Phenylalanine |
|  | L-Proline | N6,N6,N6-Trimethyl-L-lysine | L-Histidine | L-Methionine | L-Lysine | Beta-Citryl-L-glutamic acid |
|  | L-Phenylalanine | L-Valine | L-Phenylalanine | L-Arginine | L-Histidine | N-Acetyl-L-methionine |
|  | Beta-Citryl-L-glutamic acid | L-Methionine | L-Tyrosine | L-Aspartic acid | L-Tyrosine | L-Methionine |
|  | N6,N6,N6-Trimethyl-L-lysine | L-Glutamine | D-Leucine | L-Histidine | L-Glutamic acid | L-Lysine |
|  | L-Methionine | L-Arginine | Beta-Leucine | L-Proline | N-Acetylglutamic acid | L-Asparagine |
|  | L-Cystine | L-Proline | N-Acetylglutamic acid | L-Phenylalanine | Kynurenic acid | L-Threonine |
|  | L-Aspartic acid | L-Tyrosine | Kynurenic acid | N-Acetylglutamic acid | L-Tryptophan | L-alpha-glutamyl-L-hydroxyproline |
|  | L-Histidine | L-gamma-glutamyl-L-valine | L-Leucine | L-Leucine | L-Methionine | o-Tyrosine |
|  | L-Asparagine | D-Lysine | L-Alloisoleucine | D-2-Hydroxyglutaric acid | L-Arginine | Beta-Tyrosine |
|  | L-Tyrosine | Kynurenic acid | L-Isoleucine | L-Isoleucine | L-Histidine | Kynurenic acid |
|  | L-Glutamic acid | L-Leucine | N-Acetylhistamine | L-Glutamine | L-Phenylalanine | L-Leucine |
|  | N-Acetylglutamic acid | L-Lysine | L-Glutamine | L-Arginine | L-Tyrosine | Pyroglutamic acid |
|  | Kynurenic acid | L-Isoleucine | L-Arginine | L-Histidine | N-Lauroylglycine | L-Isoleucine |
|  | L-Leucine | N-Acetylhistamine | L-Tyrosine | L-Histidine | Glutarylglycine | L-Tyrosine |
|  | L-Isoleucine | o-Tyrosine | Tridecanoylglycine | L-Phenylalanine | Palmitoylglycine | L-Glutamic acid |
|  | L-Arginine | Beta-Tyrosine | N-Undecanoylglycine | L-Tyrosine | N-Undecanoylglycine | Urocanic acid |
|  | L-Glutamic acid | L-Tyrosine | N-Nonanoylglycine | L-Glutamic acid | N-Decanoylglycine | L-Cysteinylglycine disulfide |
|  | L-Lysine | Pristanoylglycine | N-Decanoylglycine | L-Tryptophan | Tridecanoylglycine | Cysteineglutathione disulfide |
|  | L-Tyrosine | N-Decanoylglycine | Pristanoylglycine | L-2-Hydroxyglutaric acid | Pristanoylglycine | Ornithine |
|  | D-Tryptophan | Myristoylglycine | Margaroylglycine | 3-Hydroxyglutaric acid | Pentacosanoylglycine | Dimethylglycine |
|  | L-Tryptophan | Palmitoylglycine | Methylglutaric acid | N-Undecanoylglycine | N-Nonanoylglycine | Tridecanoylglycine |
|  | Pristanoylglycine | N-Undecanoylglycine |  | N-Decanoylglycine | Myristoylglycine | Pristanoylglycine |
|  | N-Decanoylglycine | Tridecanoylglycine |  | Tridecanoylglycine | Lipoyllysine | N-Undecanoylglycine |
|  | Myristoylglycine | N-Nonanoylglycine |  | Pristanoylglycine | Ornithine | N-Nonanoylglycine |
|  | Palmitoylglycine | N-Lauroylglycine |  | Palmitoylglycine |  | N-Decanoylglycine |
|  | Glutarylglycine | Lipoyllysine |  | Glutarylglycine |  | Myristoylglycine |
|  | N-Undecanoylglycine | Ornithine |  | N-Nonanoylglycine |  | N-Lauroylglycine |
|  | Tridecanoylglycine | Dimethylglycine |  | N-Lauroylglycine |  | Palmitoylglycine |
|  | N-Nonanoylglycine |  |  | Lipoyllysine |  | Lipoyllysine |
|  | Lipoyllysine |  |  | Ornithine |  |  |
|  | Ornithine |  |  | Dimethylglycine |  |  |
|  | Dimethylglycine |  |  | 3-Methylglutaconic acid |  |  |
|  | Hippuric acid |  |  | Urocanic acid |  |  |
|  | 3-Methylglutaconic acid |  |  |  |  |  |
| **peptides** | Histidinyl-Histidine | Histidinyl-Histidine | L-Cysteinylglycine disulfide | Cysteinyl-Cysteine | Glutamyl-Glutamate | Histidinyl-Histidine |
|  | L-Cysteinylglycine disulfide | Glutamyl-Glutamate | Glutarylglycine | Glutamyl-Glutamate | Cysteinyl-Cysteine | L-Cysteinylglycine disulfide |
|  | Cysteinyl-Cysteine | L-Cysteinylglycine disulfide | Arginyl-Arginine | L-Cysteinylglycine disulfide | Gamma-Glutamyltyrosine | L-Threo-3-Phenylserine |
|  | Oxidized glutathione | Leucyl-Aspartate | Carnosine | Asparaginyl-Arginine | L-Cysteinylglycine disulfide | Glutarylglycine |
|  | Glutathione | Isoleucyl-Aspartate | Oxidized glutathione | Arginyl-Asparagine | Oxidized glutathione | Cysteinyl-Cysteine |
|  | Cysteineglutathione disulfide | Hydroxyprolyl-Cysteine |  | Carnosine |  | Arginyl-Arginine |
|  |  | Cysteinyl-Hydroxyproline |  | Oxidized glutathione |  | Oxidized glutathione |
|  |  | Aspartyl-Leucine |  | Glutathione |  |  |
|  |  | Aspartyl-Isoleucine |  | L-Cystathionine |  |  |
|  |  | L-beta-aspartyl-L-leucine |  |  |  |  |
|  |  | Arginyl-Arginine |  |  |  |  |
|  |  | Oxidized glutathione |  |  |  |  |
|  |  | L-Cystathionine |  |  |  |  |
| **acylcarnitines** | Hydroxybutyrylcarnitine | L-Acetylcarnitine | Pimelylcarnitine | Hydroxybutyrylcarnitine | Pimelylcarnitine | L-Acetylcarnitine |
|  | 9,12-Hexadecadienoylcarnitine | Pimelylcarnitine | 2-trans4-cis-Decadienoylcarnitine | L-Acetylcarnitine | 2-trans4-cis-Decadienoylcarnitine | 3-Hydroxy-9-hexadecenoylcarnitine |
|  | 3-Hydroxy-9-hexadecenoylcarnitine | 2-trans4-cis-Decadienoylcarnitine | 6-Keto-decanoylcarnitine | 9,12-Hexadecadienoylcarnitine | 6-Keto-decanoylcarnitine | 2-trans4-cis-Decadienoylcarnitine |
|  | Pimelylcarnitine | 6-Keto-decanoylcarnitine | 12-Hydroxy-12-octadecanoylcarnitine | Pimelylcarnitine | 12-Hydroxy-12-octadecanoylcarnitine | 6-Keto-decanoylcarnitine |
|  | 2-trans4-cis-Decadienoylcarnitine | 12-Hydroxy-12-octadecanoylcarnitine | Hydroxybutyrylcarnitine | 2-trans4-cis-Decadienoylcarnitine | Butenylcarnitine | 12-Hydroxy-12-octadecanoylcarnitine |
|  | 6-Keto-decanoylcarnitine | Hydroxybutyrylcarnitine | 3-Dehydroxycarnitine | 6-Keto-decanoylcarnitine | Propenoylcarnitine | Propenoylcarnitine |
|  | 12-Hydroxy-12-octadecanoylcarnitine | Butenylcarnitine | Tiglylcarnitine | 12-Hydroxy-12-octadecanoylcarnitine | 3-Dehydroxycarnitine | 3-Dehydroxycarnitine |
|  | Butenylcarnitine | Propenoylcarnitine | Propionylcarnitine | Hydroxyvalerylcarnitine | Propionylcarnitine | Propionylcarnitine |
|  | Propenoylcarnitine | 3-Dehydroxycarnitine | L-Acetylcarnitine | Butenylcarnitine | L-Acetylcarnitine |  |
|  | Tiglylcarnitine | Propionylcarnitine | L-Carnitine | Propenoylcarnitine |  |  |
|  | Propionylcarnitine | L-Carnitine |  | 3-Dehydroxycarnitine |  |  |
|  | L-Acetylcarnitine |  |  | Propionylcarnitine |  |  |
|  | L-Carnitine |  |  | L-Carnitine |  |  |
|  |  |  |  | 2-Hexenoylcarnitine |  |  |
| **eicosanoids** | Leukotriene B4 ethanolamide | Omega-Carboxy-trinor-leukotriene B4 | 18-Carboxy-dinor- Leukotriene E4 | Omega-Carboxy-trinor-leukotriene B4 | Omega-Carboxy-trinor-leukotriene B4 | 18-Carboxy-dinor- Leukotriene E4 |
|  | Omega-Carboxy-trinor-leukotriene B4 | 18-Carboxy-dinor- Leukotriene E4 | 13E-Tetranor-16-carboxy- Leukotriene E4 | 18-Carboxy-dinor- Leukotriene E4 | 18-Carboxy-dinor-leukotriene E4 | Leukotriene B4 ethanolamide |
|  | 18-Carboxy-dinor- Leukotriene E4 | 13E-Tetranor-16-carboxy- Leukotriene E4 | Omega-Carboxy-trinor-leukotriene B4 | 13E-Tetranor-16-carboxy- Leukotriene E4 | 13E-Tetranor-16-carboxy- Leukotriene E4 | 67-dihydro-5-oxo-12-epi- Leukotriene B4 |
|  | 13E-Tetranor-16-carboxy- Leukotriene E4 | Leukotriene B4 ethanolamide | 6,7-dihydro-12-epi- Leukotriene B4 | Leukotriene B4 ethanolamide | Leukotriene B4 ethanolamide | 5-Oxo-6-trans-leukotriene B4 |
|  | Leukotriene B4 ethanolamide | 5-Oxo-6-trans-leukotriene B4 | 10,11-dihydro-leukotriene B4 | 10,11-dihydro-12-oxo- Leukotriene B4 | 13,14-Dihydro Prostaglandin F-1a | 1011-dihydro-12-oxo- Leukotriene B4 |
|  | 10,11-dihydro-20-dihydroxy- Leukotriene B4 | Leukotriene B5 | 12-Keto-tetrahydro-leukotriene B4 | 12(S)-Leukotriene B4 | Prostaglandin F2a ethanolamide | 12(S)-Leukotriene B4 |
|  | Prostaglandin E2 ethanolamide | 12-Keto-leukotriene B4 | Prostaglandin E2 ethanolamide | 6-trans-12-epi-Leukotriene B4 | Prostaglandin E2 ethanolamide | 6-trans-12-epi-Leukotriene B4 |
|  | 13,14-Dihydro Prostaglandin F-1a | Prostaglandin F2a ethanolamide | 13,14-Dihydro Prostaglandin F-1a | 6-trans-Leukotriene B4 | 17-HETE | 6-trans-Leukotriene B4 |
|  | Prostaglandin F2a ethanolamide | Prostaglandin E2 ethanolamide | 9,10,13-TriHOME | Leukotriene B5 | 13-HETE | Leukotriene B5 |
|  | 1314-Dihydro Prostaglandin F-1a | 13,14-Dihydro Prostaglandin F-1a | 9,12,13-TriHOME | 12-Keto-leukotriene B4 | 10-HETE | 12-Keto-leukotriene B4 |
|  | Prostaglandin G1 | bicyclo- Prostaglandin E2 | 5,6-DHET | Leukotriene B4 | 19(S)-HETE | Leukotriene B4 |
|  | 20-Hydroxy- Prostaglandin F2a | 15d Prostaglandin D2 | 11,12-DiHETrE | Omega-Carboxy-trinor-leukotriene B4 | 5-HETE | 13,14-Dihydro Prostaglandin F-1a |
|  | 9-HODE | Delta-12-Prostaglandin J2 | 8,9-DiHETrE | Prostaglandin E2 ethanolamide | 11,12-EpETrE | Prostaglandin E2 ethanolamide |
|  | 12,13-EpOME | Prostaglandin B2 | 14,15-DiHETrE | 1314-Dihydro Prostaglandin F-1a | 9-HETE | Delta-12-Prostaglandin J2 |
|  |  | Prostaglandin A2 | 17-HETE | Prostaglandin C1 | 12-HETE | Prostaglandin B2 |
|  |  | Prostaglandin J2 | 13-HETE | 9-Deoxy-delta12- Prostaglandin D2 | 12,13-DHOME | Prostaglandin B1 |
|  |  | 15-Keto-1314-dihydroprostaglandin A2 | 10-HETE | Prostaglandin-c2 | 9,10-DHOME | Prostaglandin A2 |
|  |  | 12,13-DHOME | 19(S)-HETE | bicyclo- Prostaglandin E2 | 11(R)-HETE | Prostaglandin J2 |
|  |  | 9,10-DHOME | 5-HETE | Delta-12-Prostaglandin J2 | 16(R)-HETE | Prostaglandin A1 |
|  |  |  | 11,12-EpETrE | Prostaglandin B2 | 8-HETE | 8-iso- Prostaglandin A1 |
|  |  |  | 9-HETE | Prostaglandin B1 | 15(S)-HETE | 15-Keto-1314-dihydroprostaglandin A2 |
|  |  |  | 12-HETE | Prostaglandin A2 |  | Prostaglandin F2a ethanolamide |
|  |  |  | 12,13-DHOME | Prostaglandin J2 |  | 5-HPETE |
|  |  |  | 9,10-DHOME | Prostaglandin A1 |  | 8,15-DiHETE |
|  |  |  | 11(R)-HETE | 8-iso- Prostaglandin A1 |  | 17,18-DiHETE |
|  |  |  | 16(R)-HETE | Leukotriene A4 |  | 14,15-DiHETE |
|  |  |  | 8-HETE | 15-Keto-1314-dihydroprostaglandin A2 |  | 12,13-DHOME |
|  |  |  | 15(S)-HETE | 1314-Dihydro Prostaglandin F-1a |  | 9,10-DHOME |
|  |  |  | 14R,15S-EpETrE | 12,20-DiHETE |  | 8(S)-HPETE |
|  |  |  |  | 9-HEPE |  | 11(R)-HPETE |
|  |  |  |  | 15d PGD2 |  | 11H-14,15-EETA |
|  |  |  |  | 12-KETE |  | 12(R)-HPETE |
|  |  |  |  | 5-HPETE |  | 15(S)-HPETE |
|  |  |  |  | 9-HODE |  | 12(S)-HPETE |
|  |  |  |  | 8,15-DiHETE |  |  |
|  |  |  |  | 5-KETE |  |  |
|  |  |  |  | 5,15-DiHETE |  |  |
|  |  |  |  | 17,18-EpETE |  |  |
|  |  |  |  | 17,18-DiHETE |  |  |
|  |  |  |  | 15-KETE |  |  |
|  |  |  |  | 14,15-DiHETE |  |  |
|  |  |  |  | 12-HEPE |  |  |
|  |  |  |  | 5-HEPE |  |  |
|  |  |  |  | 15H-11,12-EETA |  |  |
|  |  |  |  | 12,13-EpOME |  |  |
|  |  |  |  | 8(S)-HPETE |  |  |
|  |  |  |  | 11(R)-HPETE |  |  |
|  |  |  |  | 11H-14,15-EETA |  |  |
|  |  |  |  | 12(R)-HPETE |  |  |
|  |  |  |  | 15(S)-HPETE |  |  |
|  |  |  |  | 12(S)-HPETE |  |  |
| **steriod hormones** | Tetrahydrocorticosterone |  |  |  | 17a,21-Dihydroxypregnenolone |  |
|  |  |  |  |  | 11b,21-Dihydroxy-5b-pregnane-3,20-dione |  |
|  |  |  |  |  | 3a,21-Dihydroxy-5b-pregnane-11,20-dione |  |
|  |  |  |  |  | 3b,17a,21-Trihydroxypregnenone |  |
|  |  |  |  |  | 3b,15b,17a-Trihydroxypregnenone |  |
| **purines, pyrimidines** | Inosine triphosphate | Inosine triphosphate | Inosine triphosphate | Inosine triphosphate | Inosine triphosphate | Inosine triphosphate |
|  | 8-Hydroxy-7-methylguanine | Inosine | 8-Hydroxy-7-methylguanine | 8-Hydroxy-7-methylguanine | N4-Acetylcytidine | N4-Acetylcytidine |
|  | N4-Acetylcytidine | N4-Acetylcytidine | FAPy-adenine | N4-Acetylcytidine | FAPy-adenine | 8-Hydroxy-7-methylguanine |
|  | FAPy-adenine | FAPy-adenine | Thymidine 3,5-cyclic monophosphate | FAPy-adenine | 5-Methylthioadenosine | FAPy-adenine |
|  | 5-Methylthioadenosine | Thymidine 3,5-cyclic monophosphate | 5-Methylthioadenosine | Thymidine 3,5-cyclic monophosphate | 7-Methylguanosine | 5-Methylthioadenosine |
|  | 7-Methylguanosine | Cytidine monophosphate | 7-Methylguanosine | 5-Methylthioadenosine | 5-Methylcytosine | 7-Methylguanosine |
|  | Cytosine | 5-Methylthioadenosine | Cytosine | 7-Methylguanosine | Uridine | Cytosine |
|  | Deoxycytidine | 7-Methylguanosine | Deoxycytidine | Cytosine | 5-Fluorodeoxyuridine monophosphate | Deoxycytidine |
|  | Pseudouridine | Cytosine | Pseudouridine | Deoxycytidine | Adenine | 5-Methylcytosine |
|  | Uridine | Deoxycytidine | Uridine | Uridine |  | Uridine |
|  | Adenosine monophosphate |  | Deoxyguanosine | 5-Fluorodeoxyuridine monophosphate |  | 5-Fluorodeoxyuridine monophosphate |
|  | Adenine |  | Adenosine | Adenine |  | S-Adenosylhomocysteine |
|  |  |  | 5-Fluorodeoxyuridine monophosphate |  |  | Adenosine monophosphate |
|  |  |  | Adenosine monophosphate |  |  | Adenine |
| **other** | Arachidonoyl Serinol | Arachidonoyl Serinol | Arachidonoyl Serinol | Arachidonoyl Serinol | Arachidonoyl Serinol | Oleamide |
|  | Oleamide | Oleamide | Oleamide | Oleamide | Oleamide | Creatinine |
|  | NADH | NADH | NADH | NADH | Porphobilinogen | Creatine |
|  | Fucose 1-phosphate | Fucose 1-phosphate | S-Adenosylhomocysteine | Creatinine | Creatine | Taurine |
|  | 5-Aminolevulinic acid | S-Adenosylhomocysteine | Creatinine | Taurine | Creatinine |  |
|  | Ubiquinone-1 | Creatinine |  | Porphobilinogen |  |  |
|  | L-2-Hydroxyglutaric acid | Creatine |  | Creatine |  |  |
|  | Taurine |  |  |  |  |  |
|  | S-Adenosylhomocysteine |  |  |  |  |  |
|  | Creatinine |  |  |  |  |  |
|  | Taurine |  |  |  |  |  |
|  | Creatine |  |  |  |  |  |

KODE - oxooctadecenoic acid; DHOME – dihydroxyoctadecenoic acid; HETE - hydroxyeicosatetraenoic acid; EpETrE (EETA)- epoxyeicosatrienoic acid; HPETE – hydroperoxyeicosanoic acid; HODE - hydroxyoctadecadienoic acid; FAPy-adenine - 4,6-Diamino-5-N-formamidopyrimidine; NADH – reduced nicotinamide adenine dinucleotide.

**Table S8**: VIPs > 1 obtained from a validated PLS-DA model for lung sampling events performed during IVLP with FOLFOX. Shown are statistically significant features changing from IVLP T0 to IVLP T3. Data obtained from positive mode analysis.

| **Compound classification** | **Feature parameters** | | | | **Tentative ID** |
| --- | --- | --- | --- | --- | --- |
|  | **m/z** | **Retention time (min)** | **Adduct** | **Average VIP score** |  |
| Important features found via METLIN and annotation | 375.2164 | 13.9 | [M+H]^+^ | 28 | 16-phenyl tetranor prostaglandin E1, 8-oxo-resolvin D1 |
|  | 282.2792 | 21.4 | [M+H]^+^ | 5 | oleamide |
|  | 377.2324 | 12.9 | [M+H]^+^ | 2.1 | Resolvin D1 - D4, HDOPA |
| Other endogenous compounds found via METLIN | 247.1075 | 11.7 | [M+H]^+^ | 32 | N-acetyl-D,L-tryptophan |
|  | 166.0863 | 9.0 | [M+H]^+^ | 6.1 | L-phenylalanine |
|  | 132.102 | 7.1 | [M+H]^+^ | 4.4 | L-isoleucine, L-alanine |
|  | 205.0973 | 12.0 | [M+H]^+^ | 3.9 | L-tryptophan |
|  | 195.1189 | 5.1 | [M+H]^+^ | 2.5 | L-arginine |
|  | 323.1702 | 10.0 | [M+NH_4_]^+^ | 2.0 | Threoninyl-tryptophan |
|  | 182.0813 | 7.1 | [M+H]^+^ | 1.5 | L-Tyrosine, L-threo-3-Phenylserine, N-Hydroxy-L-phenylalanine |
|  |  |  | [M+NH_4_]^+^ |  | Phenylpyruvic acid |
|  | 104.1072 | 6.4 | [M+H]^+^ | 1.1 | Choline |

HDOPA - 11b-Hydroxy-3,20-dioxopregn-4-en-21-Oate
